# Gobbling across landscapes: Eastern wild turkey distribution and occupancy–habitat associations

**DOI:** 10.1002/ece3.8419

**Published:** 2021-12-01

**Authors:** Christopher D. Pollentier, Michael A. Hardy, R. Scott Lutz, Scott D. Hull, Benjamin Zuckerberg

**Affiliations:** ^1^ Wisconsin Department of Natural Resources Office of Applied Science Madison Wisconsin USA; ^2^ Department of Forest and Wildlife Ecology University of Wisconsin‐Madison Madison Wisconsin USA; ^3^ Present address: California Department of Fish and Wildlife Biogeographic Data Branch Sacramento California USA

**Keywords:** eastern wild turkey, gobbling survey, *Meleagris gallopavo silvestris*, occupancy modeling, spatial autocorrelation, species distribution

## Abstract

Extensive restoration and translocation efforts beginning in the mid‐20th century helped to reestablish eastern wild turkeys (*Meleagris gallopavo silvestris*) throughout their ancestral range. The adaptability of wild turkeys resulted in further population expansion in regions that were considered unfavorable during initial reintroductions across the northern United States. Identification and understanding of species distributions and contemporary habitat associations are important for guiding effective conservation and management strategies across different ecological landscapes. To investigate differences in wild turkey distribution across two contrasting regions, heavily forested northern Wisconsin, USA, and predominately agricultural southeast Wisconsin, we conducted 3050 gobbling call‐count surveys from March to May of 2014–2018 and used multiseason correlated‐replicate occupancy models to evaluate occupancy–habitat associations and distributions of wild turkeys in each study region. Detection probabilities varied widely and were influenced by sampling period, time of day, and wind speed. Spatial autocorrelation between successive stations was prevalent along survey routes but was stronger in our northern study area. In heavily forested northern Wisconsin, turkeys were more likely to occupy areas characterized by moderate availability of open land cover. Conversely, large agricultural fields decreased the likelihood of turkey occupancy in southeast Wisconsin, but occupancy probability increased as upland hardwood forest cover became more aggregated on the landscape. Turkeys in northern Wisconsin were more likely to occupy landscapes with less snow cover and a higher percentage of row crops planted in corn. However, we were unable to find supporting evidence in either study area that the abandonment of turkeys from survey routes was associated with snow depth or with the percentage of agricultural cover. Spatially, model‐predicted estimates of patch‐specific occupancy indicated turkey distribution was nonuniform across northern and southeast Wisconsin. Our findings demonstrated that the environmental constraints of turkey occupancy varied across the latitudinal gradient of the state with open cover, snow, and row crops being influential in the north, and agricultural areas and hardwood forest cover important in the southeast. These forces contribute to nonstationarity in wild turkey–environment relationships. Key habitat–occupancy associations identified in our results can be used to prioritize and strategically target management efforts and resources in areas that are more likely to harbor sustainable turkey populations.

## Graphical abstract

Extensive restoration and translocation efforts helped to reestablish eastern wild turkey populations throughout and beyond their historical range. We examined differences in wild turkey distribution and occupancy–habitat associations in two contrasting regions of Wisconsin, USA, where wild turkeys have become increasingly prevalent despite potentially limiting factors. Our findings demonstrated that the environmental constraints of turkey occupancy vary across the latitudinal gradient of the state and highlighted the plasticity of wild turkeys to various landscapes across the species’ northern range that should be incorporated in management planning.

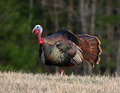

## 1 INTRODUCTION

**FIGURE 1:**
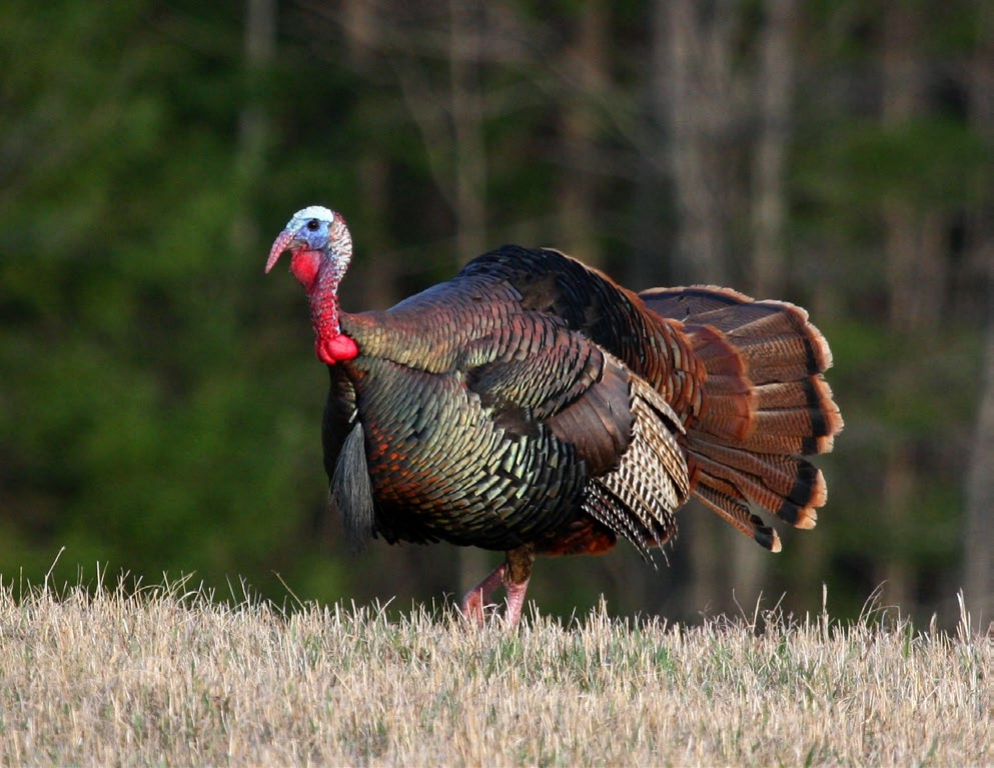
Eastern wild turkey (*Meleagris gallopavo silvestris*) occurred throughout southern Wisconsin, USA, prior to being extirpated in the late 1800s. The species now occurs statewide thanks to successful restoration efforts and rapid population expansion. Photo credit: R. S. Brady, Wisconsin Department of Natural Resources

Prior to the onset of restoration efforts in the 1960s, the prevailing belief was that eastern wild turkeys (*Meleagris gallopavo silvestris*; hereafter “turkey”; Figure 1) were unlikely to become established in the Upper Midwest of the United States due to the severity of winter weather and lack of extensive forest cover in an otherwise agriculturally dominated landscape (Porter, 2005). Initial reintroductions prioritized areas that were mostly forested, ideally with mast‐producing species such as oak (*Quercus* spp.) and hickory (*Carya* spp.), with small forest openings and nearby presence of dairy agriculture (Kubisiak et al., 2001; Wunz & Pack, 1992). Once turkeys were established within these high‐priority regions, several translocations were made to areas believed to be less suitable for turkeys, including locations with expansive forest cover where winters commonly occur with persistent deep snow, as well as rural areas that were predominately devoted to large‐scale agricultural crop production (Kubisiak et al., 2001). The successful restoration of turkeys can be attributed to these extensive translocation efforts, and the remarkable adaptability of turkeys to ever‐changing environmental conditions (Ogden, 2015) has further helped to broaden the species’ range in northern latitudes (Niedzielski & Bowman, 2015).

Today, turkeys remain of great cultural and economic significance in the United States (Chapagain et al., 2020; Isabelle et al., 2018; United States Fish & Wildlife Service, 2016). Across much of the Upper Midwest and Wisconsin, USA, abundant turkey populations are often associated with evenly mixed forest–agricultural landscapes where diverse cover types are well interspersed (Pollentier et al., 2017; Porter, 2005). However, turkeys have become established throughout Wisconsin (Dhuey & Witecha, 2020), including areas where they were once considered unlikely to persist. Populations in northern latitudes where forest cover is extensive are often limited by snow that restricts food access (Kane et al., 2007; Porter et al., 1980), resulting in lower survival when snow depth exceeds 30 cm (Lavoie et al., 2017). In southeast Wisconsin where row‐crop agriculture is the prevailing land use, turkey distribution is believed to be influenced by the dispersion and overall amount of forest cover present (Kubisiak et al., 2001). A greater understanding of turkey distributions in these regions, and how this distribution is influenced by habitat characteristics and environmental conditions, would facilitate better informed management decisions for the species.

Many approaches have been used to monitor turkey population trends and distribution, including mark–recapture (Lint et al., 1995), line or strip transects (DeYoung & Priebe, 1987), and winter flock counts (Porter & Ludwig, 1980). Wildlife management agencies often rely in part on harvest surveys and brood observation data to obtain population estimates, measure productivity, and develop management framework decisions. Although these metrics provide a valuable index of population abundance and trends over time (Healy & Powell, 1999; Lint et al., 1995), more rigorous efforts are needed to effectively investigate ecological relationships in landscapes where turkey populations are less widespread.

Gobbling call‐count surveys have been frequently used as a systematic approach to evaluate turkey distribution, population abundance, and phenology of gobbling (Bevill, 1975; Lint et al., 1995; Porter & Ludwig, 1980; Rioux et al., 2009; Scott & Boeker, 1972). However, several assumptions should be acknowledged when gobbling counts are used (Bevill, 1973; Healy & Powell, 1999), and other variables such as the chronology of breeding activity, weather conditions, and population age structure can also confound gobbling activity (Hoffman, 1990; Palmer et al., 1990; Scott & Boeker, 1972). Extrinsic factors may be difficult or impossible to control with sampling design, but when coupled with a rigorous modeling framework, gobbling call‐count surveys are capable of producing robust estimates of population status and species occurrence in relation to environmental conditions and habitat associations (Rioux et al., 2009).

Occupancy‐based models for the analysis of detection–nondetection data have been useful for evaluating population status, distributional changes, and ecological correlates of occurrence of wildlife species (MacKenzie et al., 2006). MacKenzie et al. (2002) described the initial modeling framework for estimating the probability that a site is occupied by a species given imperfect detection. Multiseason models have further permitted the investigation of site occupancy dynamics and can be used to explore how environmental factors affect occupancy rates via the ecological processes of colonization and local extinction (MacKenzie et al., 2003, 2006). Several extensions of the original static and dynamic models have since been developed to accommodate various ecological questions, address model assumptions, and offer logistical flexibility with respect to survey sampling design (Bailey et al., 2014). Turkey gobbling call‐count surveys typically consist of multiple sampling (i.e., listening) stations located at equidistant intervals along a survey route (Lint et al., 1995; Porter & Ludwig, 1980; Scott & Boeker, 1972). However, this logistical approach of conducting surveys at successive stations often yields replicates that are not independent, resulting in survey data from adjacent stations that are spatially autocorrelated. Failure to account for this spatial autocorrelation results in a lack of independence among sample data and leads to a significant bias of occupancy estimates (Hoeting, 2009; Legendre, 1993). To address the issue of spatial autocorrelation, Hines et al. (2014) developed an extension to the multiseason occupancy model of MacKenzie et al. (2003) that incorporates correlated replicates from adjacent stations along a transect‐based survey route to permit inferences about occupancy dynamics and local probabilities of extinction and colonization. The correlated‐replicate occupancy modeling approach has shown to be well‐suited for evaluating occupancy–habitat associations and spatial distributions of turkeys from gobbling call‐count survey data (Pollentier et al., 2019).

To help guide management efforts, wildlife managers and stakeholders have sought to better understand turkey distribution and habitat associations in landscapes where turkey populations have historically been less prevalent. Our primary objective was to use gobbling call‐count surveys in combination with novel multiseason correlated‐replicate occupancy models to examine the influence of habitat characteristics on the occurrence and distribution of turkey populations across 2 separate and contrasting regions of Wisconsin: (1) heavily forested northern Wisconsin and (2) agriculturally dominated southeast Wisconsin. We also evaluated the dynamic effect of winter snowfall and changes in annual agricultural cropland rotations on the establishment of unoccupied sites and abandonment of previously occupied sites. Finally, we used results from our occupancy modeling framework to identify areas of high and low occurrence probability to better assist wildlife managers and decision‐makers in prioritizing potential research, conservation, or management efforts targeting turkeys in areas with less suitable habitat and lower turkey population densities.

## 2 METHODS

### 2.1 Study area

**FIGURE 2:**
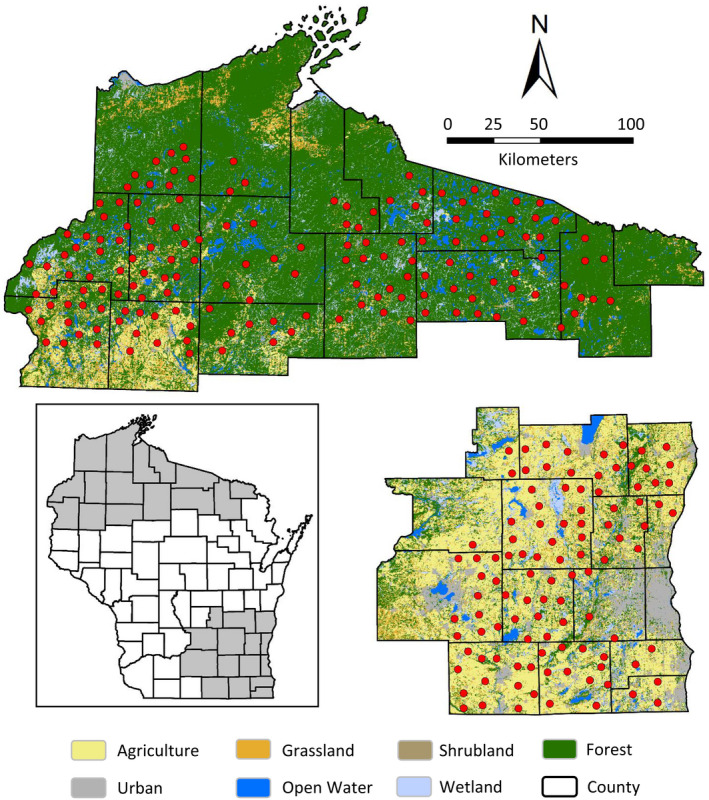
Distribution of wild turkey gobbling call‐count survey routes in northern (*n* = 157) and southeast (*n* = 103) Wisconsin, USA, 2014–2018. Inset map highlights counties (gray shaded) included in each study area. Individual points (red) indicate survey route locations. Land cover classes are shown for reference

We conducted turkey gobbling call‐count surveys across 2 contrasting regions of Wisconsin with different proportions of forest and open‐agricultural cover (Figure 2). Land cover characteristics and description of our northern Wisconsin study area are provided in greater detail elsewhere (Pollentier et al., 2019). Briefly, much of northern Wisconsin was heavily forested and largely comprised of mesic northern hardwoods of maple (*Acer* spp.) and American basswood (*Tilia americana*); scattered stands of eastern hemlock (*Tsuga canadensis*), aspen (*Populus* spp.), birch (*Betula* spp.), and pine (*Pinus* spp.); and many freshwater glacial lakes connected by meandering streams. Portions of northwest Wisconsin consisted of a mosaic of dry‐mesic pine and oak forests, barrens, and grasslands; row‐crop agriculture and dairy farming were present but limited given the coarse, sandy soils that existed. Most land in northern Wisconsin was under private ownership (approx. 62%); public land consisted of state‐ and county‐managed properties and natural areas, county forests, easements, and the Chequamegon–Nicolet National Forest managed by the United States Forest Service. Growing seasons were typically short, and cold, snowy winters were prevalent with average snowfall totals ranging from 61.0 to 353.1 cm. Turkeys were historically rare across northern Wisconsin until intrastate translocation efforts occurred during 1998–2000 (Kubisiak et al., 2001).

Survey routes across southeast Wisconsin were located within portions of the Central Lake Michigan Coastal, Southern Lake Michigan Coastal, and Southeast Glacial Plains ecological landscapes. Although much of this region could be characterized as densely populated, with nearly one‐half of the state's residents located in southeast Wisconsin, intensive row‐crop agriculture (e.g., corn, soybean, alfalfa) was the predominately land use (>60%) and created a highly fragmented landscape (Wisconsin Department of Natural Resources, 2015). The majority of land in southeast Wisconsin was privately owned (approx. 94%), and public land was mostly limited to easements, scattered state‐ and county‐managed properties, and land trusts. Upland forest cover constituted about 12% of the landscape and was generally confined to isolated patches, such as the Kettle Interlobate Moraine, where the topography was too rugged for agriculture. Wetlands also occurred on about 12% of the study area and included large marshes, sedge meadows, and forested lowlands along floodplain river bottoms. Dry mesic to mesic sites were typical of the region and often associated with loamy soils that were well drained and nutrient‐rich. Forest stands were frequently dominated by northern red oak (*Q*. *rubra*) and white oak (*Q*. *alba*), often accompanied by sugar maple (*A*. *saccharum*), white ash (*Fraxinus americana*), and American basswood. Floodplain and lowland forests were composed of a mixture of red maple (*A*. *rubrum*), green ash (*F*. *pennsylvanica*), black ash (*F*. *nigra*), and swamp white oak (*Q*. *bicolor*). Southeast Wisconsin had a continental climate, with an average minimum temperature of −14.6°C in January and an average maximum temperature of 27.3°C in August. The growing season averaged 155 days, and the mean annual precipitation was 85.3 cm. Winter snowfall totals tended to vary on a latitudinal gradient and ranged from 156.0 cm in the north to 52.8 cm toward the south. Turkeys were common in southeast Wisconsin prior to being extirpated in the late‐1880s; reintroductions of turkeys to the region began in 1979 and occurred through the mid‐1980s (Kubisiak et al., 2001).

An annual spring turkey hunting season has occurred statewide across Wisconsin since 2006. The regular spring turkey season has been comprised of six 1‐week hunting periods from mid‐April through the end of May. A youth‐only hunt has generally occurred the weekend prior to the opening of the regular season. Hunting was permitted all day, with legal hunting hours being 30 minutes before sunrise to sunset.

### 2.2 Sampling design

Turkeys can be found across a spectrum of regional environments throughout their range (Porter, 1992); in the Upper Midwest where agriculture is prominent, turkeys are often associated with small agricultural croplands that are well interspersed with forest cover (Pollentier et al., 2017; Porter, 2005). We sought to distribute our survey routes so that they were representative of each respective study area. We used ArcGIS Pro 2.3 (Environmental Systems Research Institute, Redlands, CA, USA) and Wiscland 2.0 land cover data (Wiscland; Wisconsin Department of Natural Resources, 2016) to assess land cover characteristics across 304 and 145 Public Land Survey System townships (~9300 ha each; hereafter “townships”) in northern and southeast Wisconsin, respectively. For townships in northern Wisconsin, we calculated the percentage of forest cover, which included deciduous forest, evergreen forest, mixed forest, and forested wetland, and assigned each township to 1 of 5 strata based on the proportion of forest cover (≤20% forest, >20% to ≤40% forest, >40% to ≤60% forest, >60% to ≤80% forest, and >80% forest; Pollentier et al., 2019). Our preliminary analysis of townships in southeast Wisconsin revealed that only 1 township contained >40% forest cover. Because much of the land use in this region was devoted to agricultural crop production and forest patches were generally scattered and isolated, we opted to evaluate forest patch size and categorized townships by quartiles according to low (≤6.0 ha), medium‐low (>6.0 to ≤9.8 ha), medium‐high (>9.8 to ≤25.5 ha), and high (>25.5 ha) mean forest patch size. We used a standard occupancy design to help determine the number of survey routes and annual repeat visits needed for each study area (Field et al., 2005; MacKenzie et al., 2006; MacKenzie & Royle, 2005). To infer distribution and occupancy of turkeys, we initially selected 136 gobbling call‐count survey routes in northern Wisconsin which we stratified by the number of townships in each forest cover stratum. We added an additional 19 routes to our northern study area prior to the second year of conducting surveys. Likewise, we selected 103 survey routes across southeast Wisconsin, which we categorized by mean forest patch size (Table 1). Given our survey design and modeling framework (Occupancy model development, below), we used program GENPRES (Bailey et al., 2007) to examine sampling design trade‐offs and determined that 3 annual repeat surveys in northern Wisconsin (18 days per sampling period) and 4 annual repeat visits in southeast Wisconsin (14 days per sampling period) were sufficient to achieve our objectives (Pollentier et al., 2021).

**TABLE 1:** Number of candidate Public Land Survey System townships evaluated and subsequent sample size of eastern wild turkey gobbling call‐count surveys routes in northern Wisconsin, USA, 2014–2017, and southeast Wisconsin, 2016–2018

Category^a^	Townships (*n*)^b^	Townships (%)	Survey routes (*n*)^c^
Forest stratum (N WI)			
≤20% forest cover	6	2.0	3
>20% to ≤40% forest cover	8	2.6	4
>40% to ≤60% forest cover	43	14.1	22
>60% to ≤80% forest cover	106	34.9	55
>80% forest cover	141	46.4	73
Subtotal	304	100.0	157
Mean forest patch size (SE WI)			
≤6.0 ha	37	25.5	26
>6.0 to ≤9.8 ha	36	24.8	26
>9.8 to ≤25.5 ha	36	24.8	26
>25.5 ha	36	24.8	25
Subtotal	145	100.0	103
Total	449		260

^a^ Perspective townships in northern and southeast Wisconsin study areas were categorized by percentage of forest cover and mean forest patch size (ha), respectively, and derived from Wiscland 2.0 land cover data (Wisconsin Department of Natural Resources, 2016). Forest cover included coniferous, broad‐leaved deciduous, mixed deciduous–coniferous, and forested wetlands.

^b^ Number of perspective Public Land Survey System townships (~9300 ha each) within each forest cover stratum and mean forest cover patch size category.

^c^ Total number of eastern wild turkey gobbling call‐count survey routes selected per category.

Each of our 260 survey routes consisted of 3 listening stations located at 1.6‐km equidistant intervals along secondary (i.e., paved or maintained gravel) and tertiary (i.e., dirt) roads designated for vehicle traffic. We avoided primary roadways that served as main thoroughfares, such as state and local highways or county roads, because traffic could have interfered with our ability to detect gobbling turkeys (Healy & Powell, 1999; Lint et al., 1995; Palmer et al., 1990; Porter & Ludwig, 1980; Scott & Boeker, 1972). We centered a 3.2‐km buffer (~5300 ha each) along each route and assessed percentage of forest cover and mean forested patch size to ensure routes were representative of the township where they were located. Male turkeys tend to maintain consistent home ranges during reproductive periods (Collier et al., 2017; Gross et al., 2015) despite increased daily movements within their ranges during the breeding season (Chamberlain et al., 2018; Paisley et al., 2000). Therefore, survey routes were located ≥3.2 km apart to reduce the likelihood of sampling the same individuals across multiple survey routes.

Potential biases with respect to habitat characteristics associated with gobbling surveys along roadways could occur, but we were confident our sampling design was representative of the landscape in northern and southeast Wisconsin. Both study areas had well‐developed road networks with road densities of 1.53 km/km^2^ in northern Wisconsin and 2.88 km/km^2^ in southeast Wisconsin. Additionally, gobbling turkeys can be heard from nearly 2.0 km away under favorable conditions (Healy & Powell, 1999; Rioux et al., 2009); thus, we used ArcGIS Pro 2.3 and placed 2.0‐km buffers around all secondary and tertiary roads in our study areas and found the buffers covered 98.1% of our northern study area and 99.2% of our southeast study area. Therefore, we believe our sampling framework enabled detection of turkeys away from roads and inferences would not be directly associated with conditions adjacent to roadways.

### 2.3 Gobbling call‐count surveys

We conducted roadside‐based turkey gobbling call‐count surveys in northern and southeast Wisconsin during spring 2014–2017 and 2016–2018, respectively. Surveys occurred during the final week of March through the third week of May, which corresponded to the time frame when peak gobbling activity occurred (Healy & Powell, 1999). We divided our spring surveys into sampling periods for repeat surveys, 3 in northern Wisconsin and 4 in southeast Wisconsin, as defined previously. Routes were surveyed once during each sampling period to ensure surveys were staggered across our survey window to account for daily and seasonal variation in gobbling activity and the gradual emergence of foliage throughout the spring. Prior to beginning surveys each year, surveyors were thoroughly trained in survey protocols (Pollentier et al., 2019). We drafted a survey schedule that alternated surveyors and changed the order in which routes and survey stations were visited on successive visits. Surveys were conducted 1 h before sunrise to ≤2.5 h after sunrise by a single surveyor on days without persistent precipitation and sustained wind speeds <24 km/h (i.e., ≤3 on the Beaufort scale). We performed a 4‐minute point count at each survey station and recorded all turkeys seen or heard before proceeding to the next station, and care was taken to avoid double counting of individual turkeys.

### 2.4 Environmental and land cover covariates

Several environmental variables have the potential to affect both gobbling activity and the ability of surveyors to detect turkeys (Bevill, 1973; Healy & Powell, 1999). Therefore, we recorded environmental conditions while at each station to account for factors that may influence detection probability. We recorded wind speed (km/h) and temperature (°C) immediately following completion of each 4‐min survey with a portable weather meter (Model 3500; Kestrel Instruments, Boothwyn, PA, USA). In addition, the surveyors recorded the time of day and prevailing weather conditions using categorical sky codes (0, clear or few clouds; 1, partly or variably cloudy; 2, cloudy or overcast; 3, fog or smoke; 4, drizzle; 5, rain; and 6, snow), and noted any potential noise disturbance (e.g., other bird vocalizations or passing vehicles) that could have influenced detection of turkeys by a surveyor.

We evaluated the potential influence snow cover may have on occurrence of turkeys. Particularly for turkeys across the northern extent of their range, prolonged periods of deep snow cover (>30 cm) can restrict movements and populations may experience significant overwinter losses unless reliable food sources are available (Kane et al., 2007; Porter et al., 1980; Roberts et al., 1995; Wunz & Hayden, 1975). We obtained gridded snow cover datasets from the Snow Data Assimilation System (SNODAS) via National Snow and Ice Data Center (NSIDC) and National Operational Hydrologic Remote Sensing Center (NOHRSC, 2004). The SNODAS datasets integrated snow data from satellites, airborne platforms, ground stations, and downscaled weather prediction models to create a daily snow cover map for the conterminous United States at a 30‐arc second resolution (~1 km^2^). We used SNODAS data to calculate daily snow depth (cm) across each survey route during winter (1 Nov–30 Apr) in our northern (2013–2017) and southeastern (2015–2018) study areas. For each survey route, we summed the cumulative number of days with snow depth >30 cm during each winter.

We used Wiscland to characterize land cover attributes of our gobbling survey routes and stations in northern and southeast Wisconsin. The Wiscland dataset provided a detailed land cover database with a raster resolution of 30 m; user accuracies varied across cover types (range = 17.1%–99.0%) with an overall accuracy of 72.8% (Wisconsin Department of Natural Resources, 2016). Although Wiscland contained 68 total land cover classifications, we consolidated cover classes into 14 categories according to functionality and structural characteristics that we believed were most likely to influence turkey distribution and occurrence. We reclassified land cover classes into the following categories: developed, agricultural crops, grass–pasture, mixed forest, coniferous forest, deciduous forest, aspen–birch, upland hardwoods, oak, water, wetlands, forested wetlands, barrens, and shrubland. We also combined cover classes into 2 generalized land cover categories for analysis: forest cover (all forest cover classes) and open cover (agricultural crops, grass–pasture, barrens, and shrubland cover).

Agricultural classifications within the Wiscland dataset were derived from National Agriculture Statistics Service Cropland Data Layers (CDL; United States Department of Agriculture [USDA], 2017) and aggregated across multiple years to infer land cover classification (Wisconsin Department of Natural Resources, 2016). However, row‐crop agriculture can be a dynamic cover class of various crops and often changes on an annual basis according to scheduled crop rotations. Agricultural fields, particularly corn, have been considered an important food source for turkeys across the Upper Midwest (Paisley et al., 1996; Porter, 2005) and potentially have some level of influence on turkey presence in any given year depending on the crop planted. Therefore, we opted to use annual CDL datasets to further characterize land cover classified as agriculture. The CDL for Wisconsin contained 103 unique agricultural cover classes, which we simplified for our study to evaluate the annual percentage of agriculture classified as corn (e.g., sweet corn, silage corn), grain crops (e.g., oats, wheat, other small grains), or other row crops (soybeans, vegetable crops).

We used a multiscale approach and analyzed land cover characteristics for gobbling call‐count survey stations and routes. For survey stations, we centered a 1.6‐km buffer (813.7 ha) around each of the 3 stations that comprised a route from which we assessed land cover. For survey routes, we evaluated land cover within the 3.2‐km buffer (~5300 ha) that we used to define each route. We used ArcGIS Pro 2.3 to clip Wiscland and CDL land cover raster data and used FRAGSTATS 4.2 (McGarigal et al., 2012) to assess class‐ and landscape‐level metrics of land cover composition and configuration for each survey station and route (Table 2). At the class level, we examined the percentage of land cover (PLAND) for each cover type we classified from Wiscland and CDL; we also evaluated two other metrics of cover class composition: mean patch area (AREA) and largest patch index (LPI). We examined 5 class‐level metrics of spatial context and aggregation for cover classes via the proximity index (PROX), clumpiness index (CLUMPY), interspersion and juxtaposition index (IJI), edge density (EDGE), and Euclidean nearest neighbor distance (ENN; Table 2). Open‐agricultural landscapes interspersed with forest cover have frequently been identified as suitable turkey habitat (Kurzejeski & Lewis, 1985; Paisley et al., 1996; Pollentier et al., 2017; Porter, 2005). Therefore, we also evaluated 4 configuration metrics to assess the spatial aggregation and interspersion at the landscape level (McGarigal et al., 2012): EDGE, contrast‐weighted edge density (CWED), contagion index (CONTAG), and IJI (Table 2).

**TABLE 2:** Description of land cover class‐ and landscape‐level composition and configuration metrics from FRAGSTATS 4.2 (McGarigal et al., 2012) used to assess the probability of local availability and route occupancy for eastern wild turkeys along gobbling call‐count survey stations and routes in northern Wisconsin, USA, 2014–2017, and southeast Wisconsin, 2016–2018

Spatial level^a^	Metric	Abbreviation	Units	Description
Class	Percentage of land cover^b^	PLAND	%	Percentage of land cover comprised of a corresponding cover type.
Class	Mean patch area^c^	AREA	ha	Average area of each patch comprising a landscape for a corresponding cover type.
Class	Largest patch index^c^	LPI	%	Percentage of total area comprised by the largest patch for a corresponding cover type.
Class	Clumpiness index^c^	CLUMPY	%	A measure of cover class‐specific fragmentation that is less susceptible to correlation with focal class area.
Class	Edge density^c^	EDGE	m/ha	Sum of the lengths of all edge segments for a corresponding cover type per total landscape area.
Class	Euclidean nearest neighbor distance^c^	ENN	m	Average shortest straight‐line distance between a focal patch and its nearest neighbor of the same cover type.
Class	Interspersion and juxtaposition index^c^	IJI	%	A measure of the extent to which a cover type is interspersed and adjacent to other cover types.
Class	Proximity index^c^	PROX	None	A measure of patch isolation and degree of fragmentation of corresponding patch types within a specified search radius (300 m).
Landscape	Edge density	EDGE	m/ha	Total sum of the lengths of all edge segments in a landscape.
Landscape	Contrast‐weighted edge density^d^	CWED	m/ha	A standardized measure of the length of each edge segment proportionate to the corresponding contrast weight between adjacent cover types.
Landscape	Contagion index	CONTAG	%	A measure of spatial dispersion and extent to which cover types are aggregated.
Landscape	Interspersion and juxtaposition index	IJI	%	A measure of the distribution of adjacencies among unique patch types.

^a^ Level of spatial heterogeneity defining landscape metrics, where class‐level metrics are integrated over all the patches of a given type (class), and landscape‐level metrics are integrated over all patch types or classes over the full extent of the data (i.e., the entire landscape; McGarigal et al., 2012).

^b^ Metric used to evaluate reclassified land cover classes from Wiscland 2.0 land cover data (Wisconsin Department of Natural Resources, 2016): developed, agricultural crops, grass–pasture, mixed forest, coniferous forest, deciduous forest, aspen–birch, upland hardwoods, oak, water, wetlands, forested wetlands, barrens, shrubland, and 2 generalized cover classes of forest cover forest cover (deciduous forest, mixed forest, evergreen forest, and forested wetland) and open cover (agricultural crops, grass–pasture, barrens, and shrubland cover). We also estimated the percentage of agriculture planted in corn (e.g., sweet corn, silage corn), grain crops (e.g., oats, wheat, other small grains), and other row crops (soybeans, vegetable crops) from Cropland Data Layers (United States Department of Agriculture [USDA], 2017).

^c^ Metric used to evaluate reclassified land cover classes from Wiscland 2.0 land cover data: developed, agricultural crops, grass–pasture, mixed forest, coniferous forest, deciduous forest, aspen–birch, upland hardwoods, oak, water, wetlands, forested wetlands, barren, and shrubland.

^d^ Maximum contrast values were assigned between forests and open‐agricultural cover classes and assigned lower values between edges of other cover classes (i.e., edge between evergreen and deciduous forest).

### 2.5 Occupancy model development

The basic sampling scheme for turkey gobbling call‐count surveys entails sampling along survey routes, where each route has multiple spatial replicates (e.g., survey stations along a road) that are surveyed sequentially. The multiseason correlated‐replicate occupancy model (Hines et al., 2014) lends itself well to such transect‐based sampling designs, including our gobbling call‐count survey data (Pollentier et al., 2019), as it accounts for potential underlying spatial autocorrelation among adjacent survey stations and allows for quantification of detection–environmental associations. Correlated‐replicate occupancy models are comprised of similar parameters as standard multiseason occupancy models, including initial occupancy ($\psi_{i}$), local extinction ($\varepsilon_{i}$), and colonization ($\gamma_{i}$), that describe transitions in the occupancy status of a route (*i*) over a specified time period such as seasons or years (MacKenzie et al., 2003). To reflect the description of potential turkey movements between temporally adjoining sampling periods estimated by $\varepsilon_{i}$ and $\gamma_{i}$, we refer to these rates as “abandonment” and “establishment,” respectively. We caution that these changes may not always correspond to actual “abandonment” and “establishment” of a route by turkeys, but instead may reflect variation in gobbling activity. The detection process is not directly analogous to the detection probability of standard occupancy modeling, as it is divided into 2 components: (1) probability of the presence at a station (*j*) given the species of interest is unavailable ($\theta_{\mathrm{ij}}$) or available ($\theta_{\mathrm{ij}}^{\prime }$) at the previous station (*j*−1), and (2) probability of detection ($p_{\mathrm{ij}}$) given the presence at a station (Hines et al., 2014). Finally, we note that at the first station surveyed along a route, there is no prior station (*j*−1) from which the probability of availability can be inferred. Therefore, we defined $\pi_{i}$ as the probability of availability at an unobserved station prior to the first survey station and fixed the estimate of $\pi_{i}$ by the Markov equilibrium process (Hines et al., 2010, 2014); thus, turkeys would be equally likely to be available at an unobserved station as at other stations.

The correlated‐replicate model allows for inference at 2 different scales: the survey route ($\psi_{i}$) and survey stations along the route ($\theta_{\mathrm{ij}}$and $\theta_{\mathrm{ij}}^{\prime }$). Therefore, we adopted the terms “occupied” to describe when turkeys were present on a route and “available” to describe when turkeys were present at a specific station to distinguish between these 2 scales of inference (Nichols et al., 2009). The data underlying our occupancy model were the detection histories for multiple seasons, where turkey(s) were either detected (1) or not detected (0). Inference is based on the set of station‐specific detection histories for all sampled routes, and model likelihood is obtained as the product of the probabilities of all observed detection histories (Hines et al., 2014). Each parameter in the likelihood can be modeled as functions of route‐ (*i*) and season‐ (*t*) specific covariates, and parameters associated with the detection process can also be attributed to station‐specific (*j*) covariates (MacKenzie et al., 2006). The maximum‐likelihood estimation can then be implemented (as in Program PRESENCE; Hines, 2006) to assess model fit and obtain parameter estimates.

**FIGURE A1:**
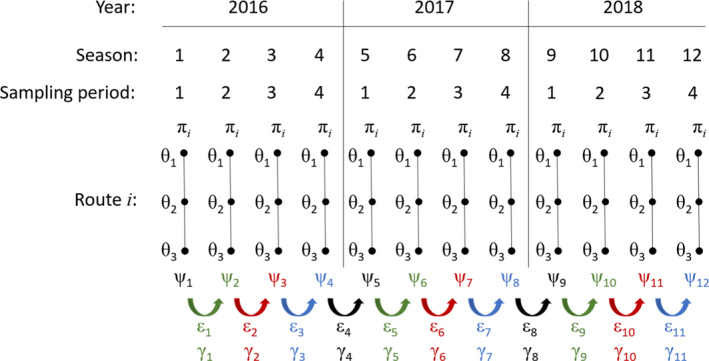
Illustration of how eastern wild turkey gobbling call‐count survey data from southeast Wisconsin, USA, 2016–2018, was coded in the design matrix of Program PRESENCE v12.23 (Hines, 2006) following the framework of Pollentier et al. (2019). We used the multiseason correlated‐replicate occupancy model and coded our data as 12 seasons, where each of the 3 survey years had 4 sampling periods, and each route had 3 survey replicates per season (i.e., 3 survey stations per route) where wild turkeys were either detected (1) or undetected (0). $\pi_{i}$ is the probability of availability at the unobserved station given route *i* is occupied. Probability of local availability ($\theta_{j}$) is estimated for each station (*j*) given route *i* is occupied and accounts for whether wild turkeys were available for detection at the previous station. Route occupancy ($\psi_{\mathrm{it}}$), abandonment ($\varepsilon_{\mathrm{it}}$), and establishment ($\gamma_{\mathrm{it}}$) are estimated for each season (*t*), and within‐year changes in occupancy could occur between the first and second sampling period (green shaded), second and third sampling period (red shaded), and third and fourth sampling period (blue shaded). Between survey years (black), $\psi_{\mathrm{it}}$, $\varepsilon_{\mathrm{it}}$, and $\gamma_{\mathrm{it}}$ represent probability of occupancy, abandonment, and establishment between 2016–2017 and 2017–2018, respectively

Occupancy models require several critical assumptions, including no unmodeled heterogeneity, independent survey outcomes, species are not misidentified or falsely detected when absent, and the population is closed to within‐season additions or losses (MacKenzie et al., 2006). We were able to satisfy most of these assumptions via our sampling design, evaluation of potential covariates, and use of the correlated‐replicate modeling approach to account for autocorrelation between survey stations. However, turkeys are highly mobile, and we remained concerned about violating the within‐season closure assumption, which could impart bias in estimates of occupancy and detection (Hayes & Monfils, 2015). Each year, our surveys were conducted over the course of defined sampling periods (3 periods in our northern study region and 4 periods in our southeast region) to account for temporal changes in gobbling activity. Thus, to address our concerns regarding the within‐season closure assumption, we coded our dataset to have discrete within‐season intervals in Program PRESENCE 12.23 and treated each of the 3 stations within a survey route as a spatial replicate (Pollentier et al., 2019; Figure A1). Under this scenario, we made no assumption of closure over sampling periods within a given year; instead, seasonal (*t*) changes in occupancy could occur between sampling periods for each route (*i*) via abandonment or establishment.

### 2.6 Data analysis

#### 2.6.1 Modeling approach

Our primary objective was to examine the influence of environmental and land cover variables on occupancy and distribution of turkeys in contrasting regions between northern and southeast Wisconsin. Prior to developing our model sets, we standardized all covariates and assessed multicollinearity among potential covariates for each model parameter with Pearson's correlation coefficients (*r*) and limited multiple variables within individual models to those where $\left|r\right|$ < 0.60 and we deemed to be biologically plausible (Dormann et al., 2013). We considered main effect models and multicovariate models with additive (+) and interactive (×) effects, and we also considered potential quadratic effects to assess nonlinear responses. We used Program PRESENCE 12.23 to build and evaluate multiseason correlated‐replicate occupancy models using Akaike's information criterion adjusted for small sample sizes (AIC*_c_*) in an information‐theoretic framework (Burnham & Anderson, 2002). We developed a suite of a priori models and conducted our analyses using an iterative approach by retaining the best‐supported model(s) within a model set (ΔAIC*_c_* < 2) for use as the base model(s) for the subsequent model set.

Our initial model set evaluated the potential influence of several covariates on gobbling activity and the ability of surveyors to detect turkeys, including time of day (where sunrise = 0 and minutes before or after sunrise are negative or positive values, respectively), temporal effects (year, sampling period, date), environmental conditions (wind speed, cloud cover, temperature, precipitation), and noise disturbance (e.g., other bird vocalizations or passing vehicles). Even though we took steps to reduce surveyor bias via trainings and alternating successive survey visits, some surveyors may have been more apt at detecting turkeys than others, so we also included a model to evaluate surveyor effect. From this initial model set, we identified the best‐supported model(s) for detection probability while holding all other model parameters constant (e.g., *ψ*[.], *θ*[.], *θ*′[.], *γ*[.], *ε*[.]). We then continued our iterative approach and built upon the best‐supported model(s) for detection probability to evaluate the influence of land cover characteristics on local turkey availability within 1.6‐km buffers centered on survey stations and initial occupancy of turkeys within 3.2‐km buffers encompassing survey routes. Our final model set focused on parameters governing the ecological dynamic processes of route occupancy influenced by establishment and abandonment. We expected probabilities of establishment and/or abandonment to vary given the annual percentage of agricultural cropland planted in corn, small grain, or other row crops. Likewise, we hypothesized that the amount of winter snow cover (measured as the number of days with >30 cm of snow) could hinder turkey movements, or may contribute to overwinter mortality in cases of prolonged deep snow cover, and thus influence probabilities of route establishment and abandonment.

Our multistage model selection strategy could be susceptible to misinterpretation of important covariates if top‐ranked model(s) were not accurately identified in any one submodel set (Morin et al., 2020). However, we carefully considered the suite of potential covariates and combinations for each model parameter and built models to represent competing a priori hypotheses (Burnham & Anderson, 2002) to efficiently explore land cover characteristics that potentially influence turkey occupancy and distribution in contrasting regions of Wisconsin. Upon completion of our final model sets, we derived model estimates from the minimum AIC*_c_* model or by model‐averaging via Akaike weights ($w_{i}$) if multiple models were equally parsimonious (ΔAIC*_c_* values <2; Burnham & Anderson, 2002). We assessed the importance of covariates for each model parameter by calculating the absolute value of *β*/SE and assessing 90% confidence intervals (Arnold, 2010; Pagano & Arnold, 2009). We assumed covariate estimates with 90% confidence intervals that did not include 0 influenced detection, local availability, route occupancy, or establishment and abandonment probabilities, whereas confidence intervals that included 0 did not influence these probabilities. Parameter probabilities and covariate beta estimates from best‐supported models are presented with ± standard error (SE).

#### 2.6.2 Predicted probability of occupancy

After we assessed our final model sets, we employed the best‐supported model from each study area to predict the probability of turkey patch occupancy across northern and southeast Wisconsin, respectively (Kéry et al., 2010). To predict occupancy probability for areas beyond our survey routes, we delineated habitat patches across each study region by dividing townships into nine township blocks and identified the centroid within each block. We then centered a ~5300 ha buffer around each centroid, which was consistent with our scale of survey route selection, and calculated land cover variables within each of these buffers in northern (*n* = 4127 buffers) and southeast (*n* = 2393 buffers) Wisconsin, respectively. Using the best‐fitting models, we predicted the patch‐specific probability of occupancy given land cover characteristics for each buffer and projected wild turkey distribution across each of our study regions.

## 3 RESULTS

**FIGURE A2:**
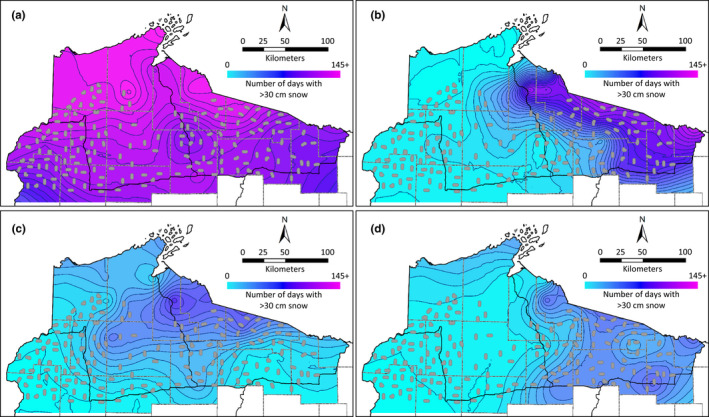
Estimated number of days with >30 cm snow cover during winter (November 1–April 30) in (a) 2013–2014, (b) 2014–2015, (c) 2015–2016, and (d) 2016–2017 in northern Wisconsin, USA. Estimates were derived from daily gridded snow cover data from the Snow Data Assimilation System via National Snow and Ice Data Center and National Operational Hydrologic Remote Sensing Center (NOHRSC, 2004). Contour lines represent 5‐day increments in number of days with >30 cm of snow cover. County boundaries (gray lines), turkey management zones (black lines), and survey routes (gray ellipses) are shown for reference

During March–May of 2014–2017, we conducted 1815 surveys (*n* = 406, 471, 471, and 467) across 157 gobbling call‐count routes in northern Wisconsin and detected turkeys on 137 routes; detections over multiple sampling periods occurred on 103 routes. The average number of days during winter (November 1–April 30) with snow depth >30 cm varied considerably across the study area and by year (*F*_3,624_ = 821.5, *p* ≤ .001; Figure A2), with the highest average occurring in winter 2013–2014 (114.0 ± 1.10 [SE] days). In each of the subsequent years we conducted surveys in northern Wisconsin, the number of days with snow depth >30 cm averaged 28.3 ± 2.69, 14.5 ± 1.35, and 12.1 ± 1.06 during winter 2014–2015, 2015–2016, and 2016–2017, respectively. On average, corn and small grain crops made up 38.60% (SE = 1.00) and 13.20% (SE = 0.78), respectively, of agricultural cover within our northern Wisconsin survey routes.

**FIGURE A3:**
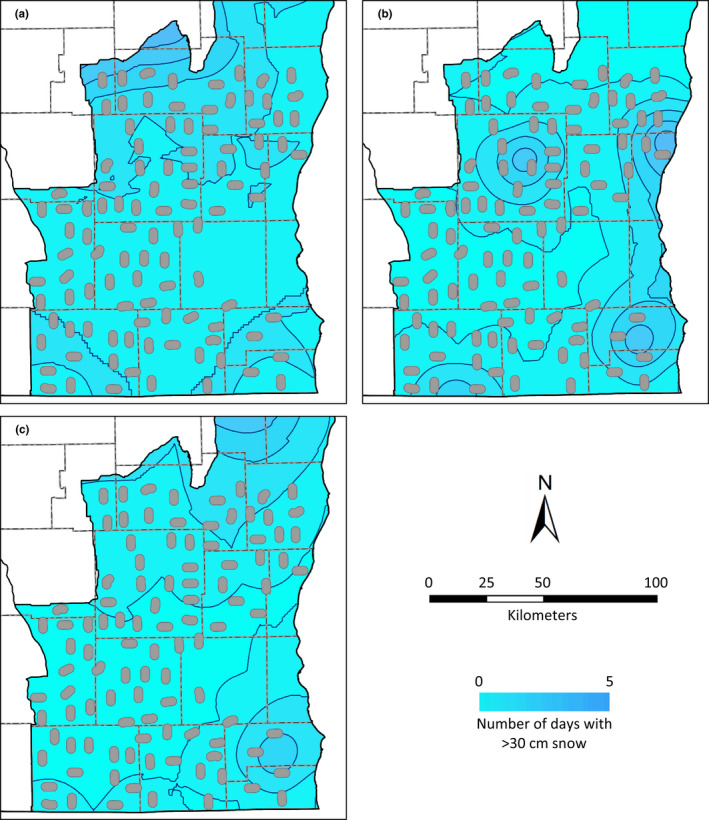
Estimated number of days with >30 cm snow cover during winter (November 1–April 30) in (a) 2015–2016, (b) 2016–2017, and (c) 2017–2018 in southeast Wisconsin, USA. Estimates were derived from daily gridded snow cover data from the Snow Data Assimilation System via National Snow and Ice Data Center and National Operational Hydrologic Remote Sensing Center (NOHRSC, 2004). Contour lines represent 1‐day increments in number of days with >30 cm of snow cover. County boundaries (gray lines), turkey management zones (black lines), and survey routes (gray ellipses) are shown for reference

In southeast Wisconsin, we performed 1235 surveys (*n* = 411, 412, and 412) on 103 routes during March–May of 2016–2018 and detected turkeys on all but 4 routes; we detected male turkeys over multiple sampling periods on 89 routes. Snowfall in southeast Wisconsin was minor relative to northern Wisconsin; the overall average number of days with snow depth >30 cm during winter 2015–2016, 2016–2017, and 2017–2018 was fewer than 1 day ($\bar{x}$ = 0.43, range = 0–2 days; Figure A3). Corn and small grain crops constituted on average 50.23% (SE = 0.49) and 5.09% (SE = 0.18), respectively, of the agricultural land cover within our southeast Wisconsin survey routes.

### 3.1 Detection and local availability

**FIGURE 3:**
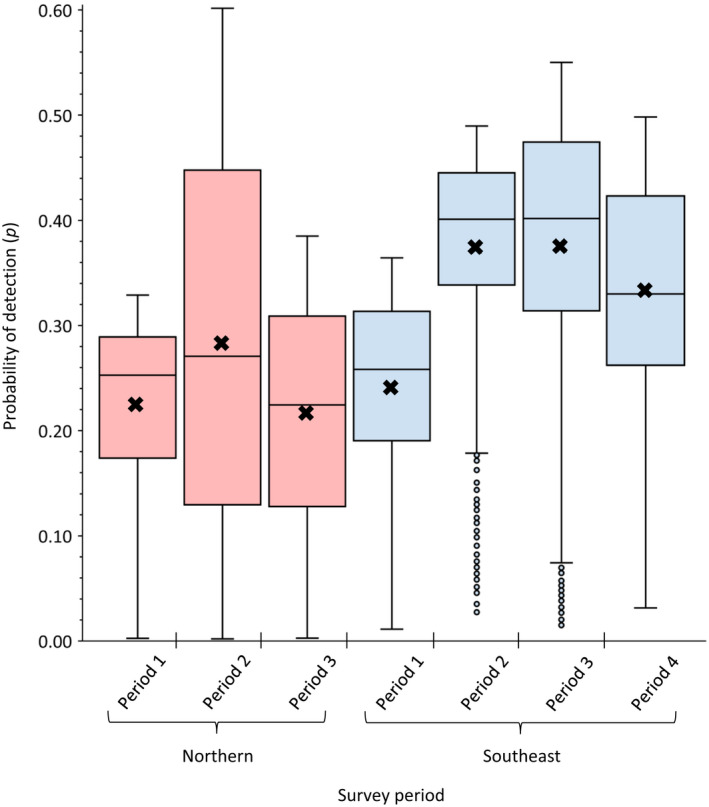
Detection probability (*p*) of eastern wild turkeys by survey period at gobbling call‐count survey stations in northern (red) and southeast (blue) Wisconsin, USA, 2014–2017 and 2016–2018, respectively. In northern Wisconsin, surveys occurred within each period during approximately March 27–April 13 (*n* = 1810), April 14–May 1 (*n* = 1815), and May 2–May 19 (*n* = 1818) for periods 1–3, respectively. In southeast Wisconsin, surveys occurred within each period during approximately March 27–April 9 (*n* = 927), April 10 –April 23 (*n* = 927), April 24–May 7 (*n* = 927), and May 8–May 21 (*n* = 924) for periods 1–4, respectively. Solid horizontal lines represent medians, crosses represent survey period means, boxes delineate interquartile ranges (IQRs), and boxplot whiskers delineate IQR boundary values (±1.5 × IQR). Individual points represent outlying absolute values greater than 1.5 × IQR

**FIGURE 4:**
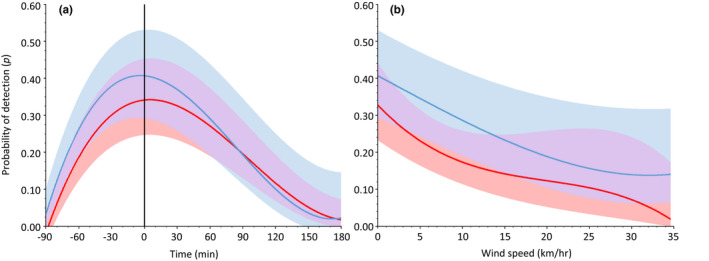
(a) Influence of the time of day (minutes before or after sunrise, and vertical line represents sunrise at 0 min) and (b) wind speed (km/h) on the probability of detecting male eastern wild turkeys during 8‐week spring (late Mar to mid‐May) gobbling call‐count surveys in northern Wisconsin, USA, 2014–2017 (red trendline), and southeast Wisconsin, 2016–2018 (blue trendline). Maximum‐likelihood estimates of detection probability were derived from the top‐supported model (ΔAIC*_c_* < 2) for northern Wisconsin (Table 3) and southeast Wisconsin (Table 4), respectively. Shaded areas represent upper and lower 95% confidence intervals for northern (light red) and southeast (light blue) study areas, and light purple shaded areas represent overlap in confidence intervals

Probability of detection varied across survey periods in northern (*F*_2,5440_ = 153.5, *p* ≤ .001) and southeast (*F*_3,3701_ = 331.7, *p* ≤ .001) Wisconsin, with detection probability highest during the second ($\hat{p}$ = .28, SE = 0.004) and third ($\hat{p}$ = 0.37, SE = 0.004) survey periods, respectively (Figure 3). Across both study areas, most (69.8%) detections occurred between 30 min prior to and 60 min after sunrise (Figure 4a) and probabilities were highest when there was little to no wind (Figure 4b). Estimated detection probabilities were highly variable across the range of survey times and wind speeds recorded during the study ($\hat{p}$ = .00 ± .002 to .60 ± .059); mean detection probability was 0.24 (SE = 0.002, *n* = 5443) in northern and 0.33 (SE = 0.002, *n* = 3705) in southeast Wisconsin, respectively.

**FIGURE 5:**
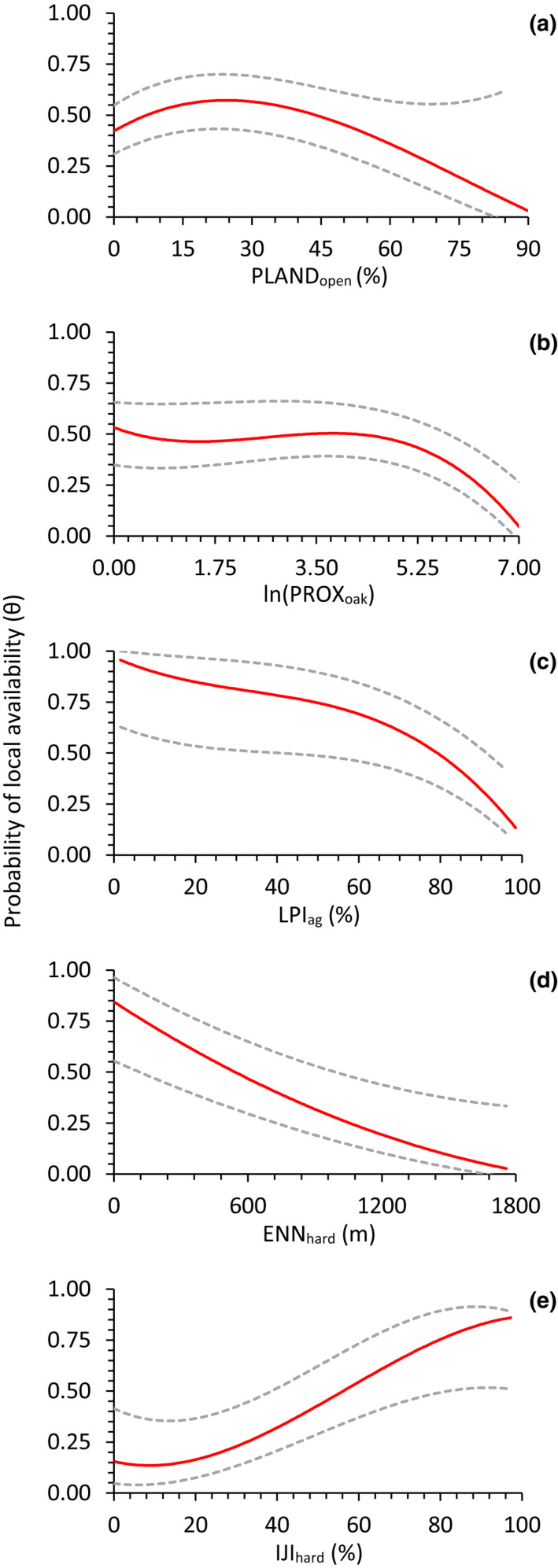
Relationships between the probability of local availability (*θ*) of eastern wild turkeys within 1.6‐km buffers around call‐count survey stations and (a) percentage of open land cover (agricultural crops, grass–pasture, barrens, and shrubland); (b) proximity of oak cover in northern Wisconsin, USA, 2014–2017; (c) largest patch index (%) of agricultural cover; (d) Euclidean nearest neighbor distance (m) of hardwood forest; and (e) interspersion and juxtaposition (%) of hardwood forest in southeast Wisconsin, 2016–2018. Maximum‐likelihood estimates of local availability were derived from the top‐supported model for northern (Table 3) and southeast Wisconsin (Table 4), respectively. Dashed lines represent upper and lower 95% confidence intervals

We found evidence of spatial autocorrelation between successive survey stations in both study areas ($\hat{\theta }$ < ${\hat{\theta }}^{\prime }$); correlation strength was stronger in the north ($\hat{\bar{\theta }}$ = 0.23 ± 0.007, $\hat{{\bar{\theta }}^{\prime }}$ = 0.73 ± 0.009) than in the southeast ($\hat{\bar{\theta }}$ = 0.47 ± 0.010, $\hat{{\bar{\theta }}^{\prime }}$ = 0.73 ± 0.009). These correlation estimates suggest that turkeys were available at an average of 46.8% (SE = 0.006) of survey stations per occupied route in northern Wisconsin and 63.6% (SE = 0.012) of survey stations per occupied route in southeast Wisconsin. Our models indicated a difference between study areas in land cover covariates that influenced the probability of local availability of turkeys. In northern Wisconsin, we found local availability was predominately influenced by the percentage of open land cover (${\text{PLAND}}_{{\text{open}}^{2}}$, $\sum w_{i}$ > 0.99; Table 3) and peaked when approximately 25% of the land cover within a 1.6‐km survey station buffer was in open cover types (Figure 5a). Proximity index of oak forest cover (${\text{PROX}}_{\text{oak}}$) was also included in our best‐supported model for northern Wisconsin (Table 3) but had only marginal influence on local availability ($\beta_{\theta }$ = −0.16 ± 0.16, $\beta_{\theta^{\prime }}$ = −0.41 ± 0.20; Figure 5b). Conversely, in southeast Wisconsin the best‐supported model within our availability model set ($w_{i}$= 0.70; Table 4) suggested that local availability was influenced by a combination of land cover metrics, including largest patch index of agriculture (${\text{LPI}}_{\text{ag}}$; $\beta_{\theta }$ = 0.08 ± 0.36, $\beta_{\theta^{\prime }}$ = −0.58 ± 0.29; Figure 5c), Euclidean nearest neighbor distance of hardwoods (${\text{ENN}}_{\text{hard}}$; $\beta_{\theta }$ = −0.81 ± 0.41, $\beta_{\theta^{\prime }}$ = −0.17 ± 0.15; Figure 5d), and interspersion and juxtaposition of hardwoods (${\text{IJI}}_{\text{hard}}$; $\beta_{\theta }$ = 1.40 ± 0.57, $\beta_{\theta^{\prime }}$ = 0.21 ± 0.40; Figure 5e).

**TABLE 3:** Multiseason correlated‐replicate occupancy model selection for eastern wild turkeys in northern Wisconsin, USA, 2014–2017

Model^a^, ^b^	*K*	AIC*_c_*	Model set ΔAIC*_c_*	Model set *w_i_*	All models ΔAIC*_c_*	All models *w_i_*
Establishment and abandonment						
*ψ*{PLAND_open_^2^ + PLAND_oak_^2^}, *θ*,*θ*′{PLAND_open_^2^ + PROX_oak_}, *γ*{C + S}, *ε*{.}, *p*{SP + (T^2^ × W)}, *π*{}	32	3119.28	0.00	0.411	0.00	0.409
*ψ*{CWED + CLUMPY_grass_}, *θ*,*θ*′{PLAND_open_^2^ + PROX_oak_}, *γ*{C^2^}, *ε*{C^2^ + G^2^}, *p*{SP + (T^2^ × W)}, *π*{}	34	3122.66	3.38	0.076	3.38	0.075
Route occupancy						
*ψ*{CWED + CLUMPY_grass_}, *θ*,*θ*′{PLAND_open_^2^ + PROX_oak_}, *γ*{.}, *ε*{.}, *p*{SP + (T^2^ × W)}, *π*{}	28	3129.22	0.00	0.275	9.94	0.003
*ψ*{PLAND_open_^2^ + PLAND_oak_^2^}, *θ*,*θ*′{PLAND_open_^2^ + PROX_oak_}, *γ*{.}, *ε*{.}, *p*{SP + (T^2^ × W)}, *π*{}	30	3130.82	1.60	0.124	11.54	0.001
*ψ*{CWED + CLUMPY_hard_}, *θ*,*θ*′{PLAND_open_^2^ + PROX_oak_}, *γ*{.}, *ε*{.}, *p*{SP + (T^2^ × W)}, *π*{}	28	3131.86	2.64	0.074	12.58	0.001
*ψ*{PLAND_oak_ + LPI_grass_}, *θ*,*θ*′{PLAND_open_^2^ + PROX_oak_}, *γ*{.}, *ε*{.}, *p*{SP + (T^2^ × W)}, *π*{}	28	3132.59	3.38	0.051	13.32	0.001
*ψ*{PLAND_open_^2^ + PLAND_oak_}, *θ*,*θ*′{PLAND_open_^2^ + PROX_oak_}, *γ*{.}, *ε*{.}, *p*{SP + (T^2^ × W)}, *π*{}	29	3132.72	3.50	0.048	13.44	0.000
*ψ*{CWED + LPI_oak_^2^}, *θ*,*θ*′{PLAND_open_^2^ + PROX_oak_}, *γ*{.}, *ε*{.}, *p*{SP + (T^2^ × W)}, *π*{}	29	3132.90	3.68	0.044	13.62	0.000
Local availability						
*ψ*{.}, *θ*,*θ*′{PLAND_open_^2^ + PLAND_oak_^2^}, *γ*{.}, *ε*{.}, *p*{SP + (T^2^ × W)}, *π*{}	28	3146.62	0.00	0.478	27.35	0.000
*ψ*{.}, *θ*,*θ*′{PLAND_open_^2^ + PROX_oak_}, *γ*{.}, *ε*{.}, *p*{SP + (T^2^ × W)}, *π*{}	26	3146.77	0.15	0.443	27.50	0.000
Detection						
*ψ*{.}, *θ*,*θ*′{.}, *γ*{.}, *ε*{.}, *p*{SP + (T^2^ × W)}, *π*{}	20	3188.23	0.000	0.979	68.95	0.000

Note

Models are ranked by the difference (ΔAIC*_c_*) between the model with the lowest Akaike's information criterion for small samples (AIC*_c_*) and AIC*_c_* for the current model, *K* is the number of model parameters, and *w_i_* is model weight. An iterative approach was used to first evaluate detection probability, and the best‐supported models (ΔAIC*_c_* < 2) were then used to sequentially assess local availability, route occupancy, and establishment and abandonment, respectively. Only models with ΔAIC*_c_* < 4 from each iterative model set are shown.

^a^ Model parameters include route occupancy (*ψ*), local availability at a survey station given unavailability (*θ*) and/or availability (*θ*′) at the previous station (*θ*), establishment (*γ*), abandonment (*ε*), detection (*p*), and availability at the unobserved survey station defined by the Markov equilibrium process via *θ* and *θ*′ (*π*). Occupancy and local availability covariates include class‐level composition and configuration metrics (McGarigal et al., 2012) for grassland–pasture (grass), oak forest (oak) and a quadratic function for oak (oak^2^), quadratic function for open cover (open^2^), and upland hardwood (hard) cover classes: clumpiness index (CLUMPY), largest patch index (LPI), percentage of land cover (PLAND), and proximity index (PROX). Contrast‐weighted edge density (CWED) between forest and open‐agricultural cover classes was also included as a landscape‐level metric. Establishment and abandonment covariates include percentage of agriculture planted in corn (C) or grain (G) and total number of days during winter (November 1–April 30) with >30 cm of snow cover (S). Detection covariates included survey period (SP), quadratic function for the number of minutes before or after sunrise (T^2^), and wind speed (W). Parameters held constant (.) within a model lack explanatory covariates.

^b^ Full model sets provided in Pollentier et al. (2021).

**TABLE 4:** Multiseason correlated‐replicate occupancy model selection for eastern wild turkeys in southeast Wisconsin, USA, 2016–2018

Model^a^, ^b^	*K*	AIC*_c_*	Model set ΔAIC*_c_*	Model set *w_i_*	All models ΔAIC*_c_*	All models *w_i_*
Route occupancy, establishment, and abandonment						
*ψ*{PROX_hard_}, *θ*,*θ*′{LPI_ag_ + ENN_hard_ + IJI_hard_}, *γ*{.}, *ε*{.}, *p*{SP + T^2^ + W}, *π*{}	28	3336.60	0.00	0.654	0.00	0.482
*ψ*{PROX_dec_}, *θ*,*θ*′{LPI_ag_ + ENN_hard_ + IJI_hard_}, *γ*{.}, *ε*{.}, *p*{SP + T^2^ + W}, *π*{}	28	3338.72	2.11	0.277	2.11	0.168
Local availability						
*ψ*{.}, *θ*,*θ*′{LPI_ag_ + ENN_hard_ + IJI_hard_}, *γ*{.}, *ε*{.}, *p*{SP + T^2^ + W}, *π*{}	27	3348.33	0.00	0.701	11.72	0.001
Detection						
*ψ*{.}, *θ*,*θ*′{.}, *γ*{.}, *ε*{.}, *p*{SP + T^2^ + W}, *π*{}	21	3405.26	0.00	0.859	68.66	0.000
*ψ*{.}, *θ*,*θ*′{.}, *γ*{.}, *ε*{.}, *p*{SP + (T^2^ × W)}, *π*{}	25	3408.93	3.67	0.137	72.32	0.000

Note

Models are ranked by the difference (ΔAIC*_c_*) between the model with the lowest Akaike's information criterion for small samples (AIC*_c_*) and AIC*_c_* for the current model, *K* is the number of model parameters, and *w_i_* is model weight. An iterative approach was used to first evaluate detection probability, and the best‐supported models (ΔAIC*_c_* < 2) were then used to sequentially assess local availability, route occupancy, and establishment and abandonment, respectively. Only models with ΔAIC*_c_* < 4 from each iterative model set are shown.

^a^ Model parameters include route occupancy (*ψ*), local availability at a survey station given unavailability (*θ*) and/or availability (*θ*′) at the previous station (*θ*), establishment (*γ*), abandonment (*ε*), detection (*p*), and availability at the unobserved survey station defined by the Markov equilibrium process via *θ* and *θ*′ (*π*). Occupancy and local availability covariates include class‐level composition and configuration metrics (McGarigal et al., 2012) for agriculture (ag), deciduous forest (dec), and upland hardwood (hard) cover classes: Euclidean nearest neighbor distance (ENN), interspersion and juxtaposition index (IJI), largest patch index (LPI), and proximity index (PROX). Detection covariates included survey period (SP), quadratic function for the number of minutes before or after sunrise (T^2^), and wind speed (W). Parameters held constant (.) within a model lack explanatory covariates.

^b^ Full model sets provided in Pollentier et al. (2021).

### 3.2 Route occupancy, establishment, and abandonment

**FIGURE 6:**
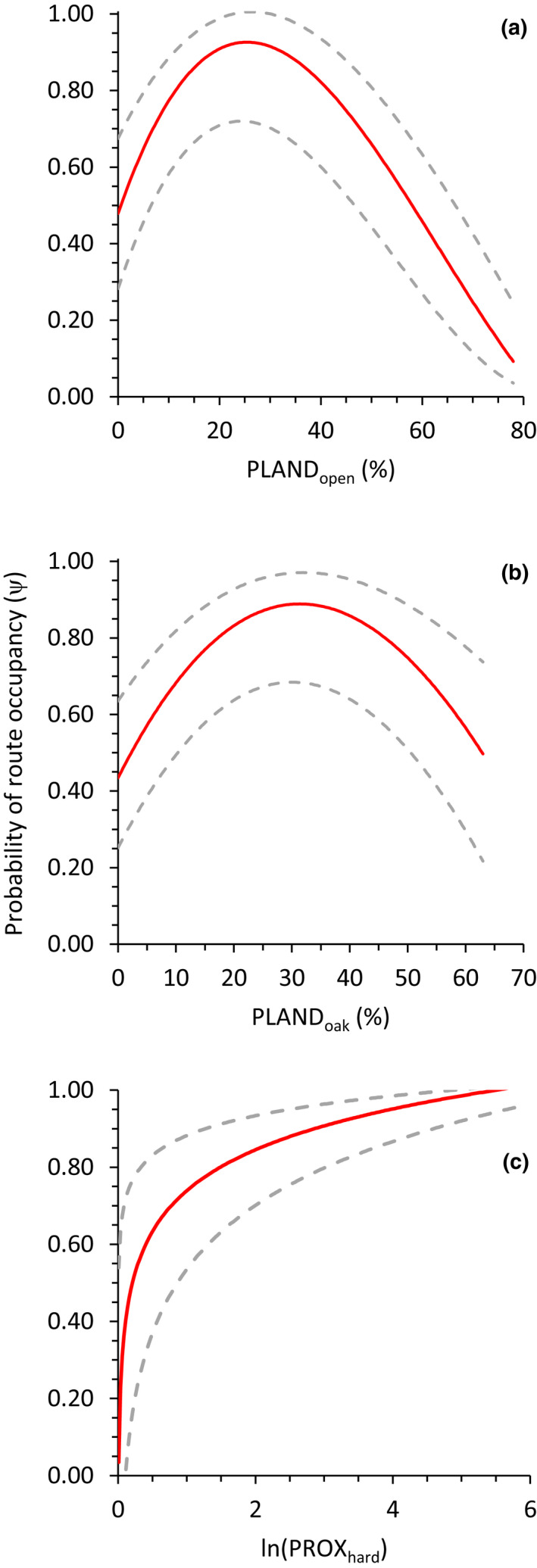
Relationship between the probability of route occupancy (*ψ*) of eastern wild turkeys within 3.2 km of call‐count survey routes (~5300 ha) and (a) percentage of open land cover (agricultural crops, grass–pasture, barrens, and shrubland); (b) percentage of oak forest cover in northern Wisconsin, USA, 2014–2017; and (c) proximity index of hardwood forest cover in southeast Wisconsin, 2016–2018. Maximum‐likelihood estimates of route occupancy were derived from the top‐supported model for northern (Table 3) and southeast Wisconsin (Table 4), respectively. Dashed lines represent upper and lower 95% confidence intervals

In northern Wisconsin, two route occupancy models were considered equally parsimonious (ΔAIC*_c_* < 2; Table 3) and both were used to further evaluate the dynamic processes of establishment and abandonment. The best‐approximating dynamic occupancy model in our final model set for northern Wisconsin (*w_i_* = 0.41; Table 3) suggested route occupancy of turkeys was most strongly influenced by a quadratic effect of percentage of open cover (${\text{PLAND}}_{{\text{open}}^{2}}$; *β* = −3.82 ± 0.14) and oak cover (${\text{PLAND}}_{{\text{oak}}^{2}}$; *β* = −1.07 ± 0.16; Table 5) within 3.2‐km route buffers. Probability of route occupancy peaked with approximately 25% of the route landscape in open cover (Figure 6a). Likewise, route occupancy tended to be highest when oak forest constituted 30%–35% of the route (Figure 6b). Our top‐supported model yielded route occupancy estimates ranging from $\hat{\psi }$ = 0.03 ± 0.019 to $\hat{\psi }$ = 0.98 ± 0.013 across survey routes in our northern Wisconsin study area during 2014–2017.

**TABLE 5:** Estimated coefficients ($\hat{\beta }$), standard errors (SE), absolute value of $\hat{\beta }/\text{SE}$, and 90% confidence intervals from the best‐supported multiseason correlated‐replicate occupancy model for eastern wild turkeys in northern Wisconsin, USA, 2014–2017, and southeast Wisconsin, 2016–2018, respectively

Covariate^a^	Study area							
	Northern				Southeast			
	$\hat{\beta }$	SE	$\left|\hat{\beta }/\text{SE}\right|$	90% CI	$\hat{\beta }$	SE	$\left|\hat{\beta }/\text{SE}\right|$	90% CI
Detection (*p*)								
${\text{Intercept}}_{1}$	−1.05	0.20		−1.39, −0.72	−0.97	0.23		−1.35, −0.59
${\text{Intercept}}_{2}$	−0.45	0.21		−0.80, −0.10	−0.25	0.26		−0.68, 0.18
${\text{Intercept}}_{3}$	−0.76	0.22		−1.12, −0.40	−0.08	0.27		−0.52, 0.37
${\text{Intercept}}_{4}$					−0.51	0.24		−0.90, −0.13
${\text{Time}}_{1}$	−0.33	0.11	2.88	−0.51, −0.14	−0.30	0.14	2.12	−0.53, −0.07
${\text{Time}}_{2}$	−0.57	0.13	4.23	−0.79, −0.35	−0.22	0.12	1.78	−0.42, −0.02
${\text{Time}}_{3}$	−0.27	0.14	2.01	−0.50, −0.05	−0.09	0.13	0.69	−0.31, 0.12
${\text{Time}}_{4}$					−0.03	0.13	0.26	−0.24, 0.18
${\text{Time}}_{1}^{2}$	−0.21	0.12	1.74	−0.42, −0.01	−0.30	0.14	2.21	−0.52, −0.08
${\text{Time}}_{2}^{2}$	−0.78	0.15	5.16	−1.03, −0.53	−0.32	0.12	2.67	−0.52, −0.12
${\text{Time}}_{3}^{2}$	−0.81	0.16	5.02	−1.08, −0.54	−0.48	0.13	3.77	−0.68, −0.27
${\text{Time}}_{4}^{2}$					−0.25	0.10	2.53	−0.41, −0.09
${\text{Wind}}_{1}$	−0.33	0.12	2.77	−0.52, −0.13	−0.31	0.14	2.21	−0.54, −0.08
${\text{Wind}}_{2}$	−0.56	0.12	4.55	−0.76, −0.36	−0.17	0.11	1.54	−0.36, 0.01
${\text{Wind}}_{3}$	−0.18	0.13	1.37	−0.40, 0.04	−0.26	0.10	2.67	−0.42, −0.10
${\text{Wind}}_{4}$					−0.48	0.14	3.36	−0.71, −0.24
${\text{Time}}_{1}$ × ${\text{Wind}}_{1}$	−0.22	0.13	1.66	−0.44, 0.00				
${\text{Time}}_{2}$ × ${\text{Wind}}_{2}$	0.41	0.14	2.85	0.17, 0.65				
${\text{Time}}_{3}$ × ${\text{Wind}}_{3}$	0.32	0.15	2.13	0.07, 0.57				
Local availability (*θ*)								
Intercept	−4.41	0.56		−5.33, −3.49	−1.65	1.52		−4.16, 0.85
${\text{PLAND}}_{\text{open}}$	−7.69	0.61	12.70	−8.69, −6.69				
${\text{PLAND}}_{{\text{open}}^{2}}$	−3.56	0.49	7.28	−4.37, −2.76				
${\text{PROX}}_{\text{oak}}$	−0.16	0.16	1.00	−0.43, 0.11				
${\text{LPI}}_{\text{ag}}$					0.08	0.36	0.23	−0.51, 0.68
${\text{ENN}}_{\text{hard}}$					−0.81	0.41	1.99	−1.47, −0.14
${\text{IJI}}_{\text{hard}}$					1.40	0.57	2.47	0.47, 2.33
Local availability (*θ*′)								
Intercept	4.46	0.61		3.65, 5.67	2.71	1.33		0.52, 4.90
${\text{PLAND}}_{\text{open}}$	6.15	0.45	13.77	5.42, 6.89				
${\text{PLAND}}_{{\text{open}}^{2}}$	2.15	0.46	4.64	1.39, 2.91				
${\text{PROX}}_{\text{oak}}$	−0.41	0.20	2.05	−0.74, −0.08				
${\text{LPI}}_{\text{ag}}$					−0.58	0.29	1.98	−1.07, −0.10
${\text{ENN}}_{\text{hard}}$					−0.17	0.15	1.14	−0.41, 0.07
${\text{IJI}}_{\text{hard}}$					0.21	0.40	0.51	−0.46, 0.87
Route occupancy (*ψ*)								
Intercept	2.72	0.45		1.97, 3.46	7.40	0.13		7.18, 7.61
${\text{PLAND}}_{\text{open}}$	−3.81	0.28	13.68	−4.27, −3.35				
${\text{PLAND}}_{{\text{open}}^{2}}$	−3.82	0.14	26.81	−4.06, −3.59				
${\text{PLAND}}_{\text{oak}}$	1.53	0.16	9.70	1.27, 1.80				
${\text{PLAND}}_{{\text{oak}}^{2}}$	−1.07	0.16	6.66	−1.33, −0.81				
${\text{PROX}}_{\text{hard}}$					26.06	0.46	56.37	25.30, 26.82
Establishment (*γ*)								
Intercept	−1.83	0.36		−2.43, −1.23	−1.60	0.39		−2.23, −0.96
Corn (%)	1.03	0.33	3.09	0.48, 1.58				
Snow	−0.93	0.49	1.89	−1.74, −0.12				
Abandonment (ε)								
Intercept	−3.85	0.91		−5.34, −2.35	−4.02	0.54		−4.92, −3.13

Note

Parameters include probability of detection (*p*), local availability at a station given unavailability (*θ*) or availability (*θ*′) at the previous station, route occupancy (*ψ*), establishment (*γ*), and abandonment (*ε*).

^a^ Detection covariates include time of day (Time), time in a quadratic form (Time^2^), average wind speed (Wind), and the interaction between time and wind (Time × Wind). Subscripts indicate survey period. Local availability covariates refer to land cover metrics within a 1.6‐km buffer around survey stations and include the percentage of land in open cover (PLAND_open_; and its quadratic form, PLAND_open_^2^), proximity index of oak forest (PROX_oak_), largest patch index of agriculture (LPI_ag_), Euclidean nearest neighbor distance of upland hardwoods (ENN_hard_), and interspersion and juxtaposition index of upland hardwoods (IJI_hard_). Route occupancy covariates refer to land cover metrics within a 3.2‐km buffer around survey routes and include percentage of land in open cover (PLAND_open_ and PLAND_open_^2^), percentage of land in oak cover (PLAND_oak_; and its quadratic form, PLAND_oak_^2^), and proximity index of upland hardwoods (PROX_hard_). Establishment covariates are defined at the route level and include the percentage of agriculture planted in corn crops (Corn [%]) and the total number of days with snow cover >30 cm (Snow) during November 1–April 30.

In southeast Wisconsin, route occupancy of turkeys was most influenced by proximity of upland hardwood forest patches (Table 4). Moreover, our best‐approximating dynamic occupancy model suggested turkey occupancy probability increased as patches of upland hardwood cover became closer and more contiguous in distribution along survey routes in southeast Wisconsin (${\text{PROX}}_{\text{hard}}$; *β* = 26.06 ± 0.46; Table 5, Figure 6c). Estimates of route occupancy ranged from $\hat{\psi }$ = 0.50 ± 0.133 to $\hat{\psi }$ = 0.99 ± 0.001 across survey routes in our southeast Wisconsin study area during 2016–2018.

**FIGURE 7:**
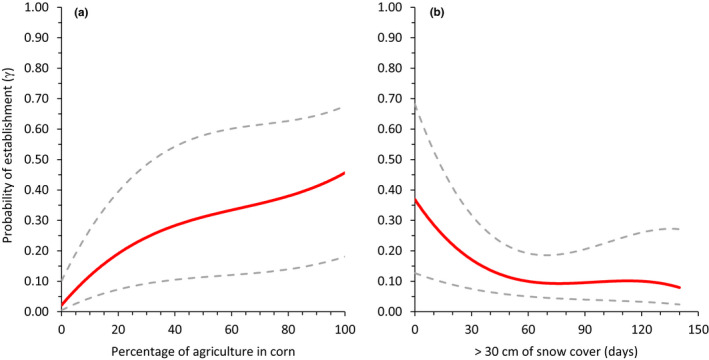
Relationship between probability of establishment (*γ*) of eastern wild turkeys and (a) percentage of row‐crop agriculture planted in corn; and (b) number of days with snow cover >30 cm from November 1–April 30 within gobbling call‐count survey routes (~5300 ha) in northern Wisconsin, USA, 2014–2017. Maximum‐likelihood estimates of route occupancy were derived from the top‐supported model (Table 3). Dashed lines represent upper and lower 95% confidence intervals

Our top‐ranked model for northern Wisconsin indicated that route establishment was positively associated with the percentage of agriculture planted in corn (*β* = 1.03 ± 0.33; Figure 7a) and negatively associated with the number of days with >30 cm of snow cover (*β* = −0.93 ± 0.49; Figure 7b). However, in southeast Wisconsin neither snow nor agricultural cover was included in our top model and establishment was best treated as a constant (Table 3) perhaps because there were so few days with persistent snow cover for inference and row‐crop agriculture is an extensive land use in the region. We were unable to find supporting evidence in either study area that abandonment of turkeys was associated with intraspecific covariates and was thus treated as a constant in our top‐ranked models for both areas (Tables 3 and 4).

### 3.3 Spatial prediction of occupancy

**FIGURE 8:**
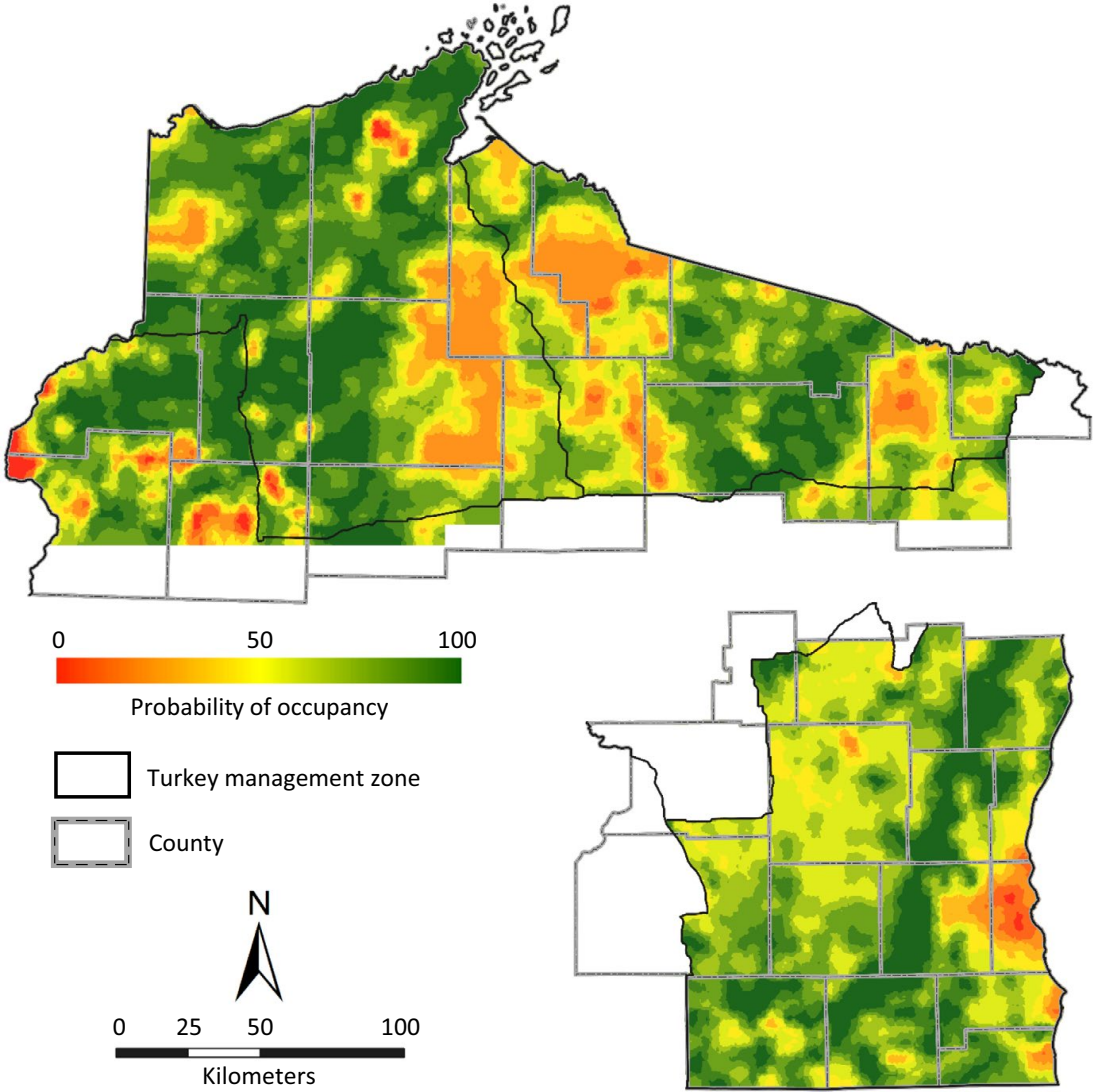
Predicted patch‐specific occupancy probability of eastern wild turkeys within northern (top) and southeast (bottom‐right) Wisconsin, USA. Spatial distribution of predicted occupancy probability for turkeys was based on predictions from the best‐supported multiseason correlated‐replicate models for each study area. In northern Wisconsin, prediction covariates included percentage of open cover and percentage of oak forest cover; and in southeast Wisconsin, the prediction covariate was proximity index of hardwood forest within a 300‐m search radius. Prediction shown is for 2017 and 2018 for northern and southeast Wisconsin, respectively, and was generated with models fit with gobbling call‐count survey data from 157 routes in northern Wisconsin and 103 routes in southeast Wisconsin. Surveys were conducted during the months of March–May of 2014–2017 in northern Wisconsin and 2016–2018 in southeast Wisconsin

Given the best‐supported models for each study area (Tables 3 and 4), probability of turkey occupancy varied substantially across northern and southeast Wisconsin, respectively (Figure 8). In northern Wisconsin, predicted estimates of patch‐specific occupancy ranged from ${\hat{\psi }}_{p}$ = 0.001 to 0.985; 26% of patches had predicted occupancy probabilities ≤50%, and 23% had a predicted occupancy ≥90%. Only 1.9% of patches were predicted to have occupancy probabilities ≤10%. Likewise, in southeast Wisconsin, predicted occupancy probabilities ranged from ${\hat{\psi }}_{p}$ = 0.001 to 0.999, but most patches (64%) had predicted occupancy probabilities between 0.50 and 0.90. Only 0.5% of patches in our southeast study region were predicted to have occupancy probabilities ≤10%, most of which occurred in heavily urbanized areas (Figure 8).

## 4 DISCUSSION

We evaluated relationships between contemporary land cover and distribution of turkeys across 2 regions of Wisconsin with contrasting landscape characteristics—heavily forested northern Wisconsin and agriculturally dominated southeast Wisconsin. We used correlated‐replicate occupancy models (Hines et al., 2010, 2014) and applied methods from a previous modeling framework evaluation of turkey gobbling call‐count surveys (Pollentier et al., 2019). The environmental constraints of turkey occupancy varied across the latitudinal gradient of the state with open land cover, snow, and row crops being relatively more important in northern Wisconsin, while the effect of hardwood forest cover was stronger in southeastern Wisconsin. Our findings suggested that, even for a habitat generalist such as the turkey (Marable et al., 2012), factors such as climate and land cover affect the occurrence of turkey populations across geographic scales (Ewers & Didham, 2006; Fahrig, 2003).

Gobbling activity peaked in mid‐ to late April, and detection probabilities were predominately influenced by time of day and wind speed at both of our study areas. We detected male turkeys throughout the morning, but most gobbling occurred near sunrise when males were likely roosting in trees, which aids sound propagation (Boncoraglio & Saino, 2007; Ey & Fischer, 2009) to attract females and maintain male dominance hierarchies (Healy, 1992; Wightman et al., 2019). High wind speeds decreased probability of detection by discouraging gobbling (Bevill, 1973), limiting the ability of surveyors to detect gobbling turkeys (Kienzler et al., 1996), or some combination thereof. Although we do not know “true” detection probability in either study system, our mean estimates ($\hat{\bar{p}}$ = .24 and 0.33 in northern and southeast Wisconsin, respectively) are consistent with previous work estimating detection probability of turkeys with repeated 10‐min point counts ($\hat{p}$ = .25; Rioux et al., 2009) and greater than estimates obtained using playback calls ($\hat{p}$ = .09–.16; Chavez, 2014; Rioux et al., 2009). Courlas (2014) estimated detection of turkeys as 0.67 with more spatial replicates (*n* = 5) per survey transect than we used (*n* = 3), but did not account for possible autocorrelation among sampling locations within transects.

Correlation strength between successive survey stations was greater in northern Wisconsin ($\hat{\bar{\theta }}$ = 0.23, $\hat{{\bar{\theta }}^{\prime }}$ = 0.73) than in southeast Wisconsin ($\hat{\bar{\theta }}$ = 0.47, $\hat{{\bar{\theta }}^{\prime }}$ = 0.73). Similar habitat between adjacent survey stations, and perhaps other endogenous factors such as movement and interactions among individuals during the reproductive season (Chamberlain et al., 2018), likely confers availability, or lack thereof, for turkeys and underscores the importance of accounting for autocorrelation within transect sampling designs (Hines et al., 2010). In northern Wisconsin, probability of local availability of turkeys peaked when ~25% of the habitat around a survey station (~814 ha) was in open cover (${\text{PLAND}}_{\text{open}}$). Meanwhile, dense clusters of large oak forest patches (${\text{PROX}}_{\text{oak}}$), which could be analogous to a large single forest‐type patch (Gustafson et al., 1994), had a negative influence on local availability of turkeys, but this relationship was marginal compared with the proportion of open cover. In southeast Wisconsin, local availability of turkeys around survey stations was negatively affected by large agricultural fields (${\text{LPI}}_{\text{ag}}$); probabilities were higher when stations contained upland hardwood forest patches that were near each other (${\text{ENN}}_{\text{hard}}$) and well interspersed (${\text{IJI}}_{\text{hard}}$). Although informative parameters of local availability differed between study areas, at this scale of inference (e.g., 1.6‐km buffers, ~814 ha) our results suggest that composition and spatial heterogeneity of diverse, contrasting land covers in otherwise forest‐ or agriculturally dominated landscapes are important factors influencing the availability of turkeys (Backs & Eisfelder, 1990; Little, 1980; Rioux et al., 2009).

Animal mobility can impart varying degrees of bias in detection, availability, and occupancy estimates, particularly in basic survey and modeling frameworks (Hayes & Monfils, 2015). Male turkeys often make frequent daily movements within their spring home ranges (Chamberlain et al., 2018; Paisley et al., 2000; Wakefield et al., 2020b), and thus may be truly unavailable for detection at a given survey station, or they may have been available but were not detected. Consequently, we implemented a sampling design at a scale to account for male turkey home range size and minimize influence of movements (Rota et al., 2009). Concerted focus of survey efforts near sunrise when detection probability was greatest for turkeys, or additional temporal replicates in combination with spatial replicates, may have improved our precision of the detection process and help to reduce potential bias of occupancy estimates in transect sampling designs (Whittington et al., 2015). However, evaluations of sampling design trade‐offs (Pollentier et al., 2019, 2021) indicated our framework was useful for decomposing the detection process into the components of local availability and detection probability given availability. Moreover, transect sampling designs have been used extensively for wildlife monitoring, and failure to account for dependence between consecutive spatial replicates has been shown to induce negative bias in occupancy estimates (Hines et al., 2010; Whittington et al., 2015). In our estimation, our survey design and modeling framework helped mitigate relative bias in occupancy estimates and our findings would be relevant for managers faced with managing landscapes and providing suitable habitat for turkeys.

Estimates of turkey occupancy probability varied considerably across survey routes in each of our study areas. In northern Wisconsin, occupancy probability was most influenced by the proportion of open cover (${\text{PLAND}}_{\text{open}}$) within survey routes (~5300 ha) and peaked when ~25% of the route buffer consisted of open cover types such as agricultural fields, herbaceous openings, and pasture–hay fields. Though less influential than open cover, proportion of oak forest cover (${\text{PLAND}}_{\text{oak}}$) was also included within our best‐supported model and probability of occupancy peaked when survey routes were composed of ~30% oak forest. Our findings were consistent with those reported by others in similar environments (Glennon & Porter, 1999; Kurzejeski & Lewis, 1985) and highlighted the benefit of open cover types for turkeys in forest‐dominated landscapes. Small scattered herbaceous openings or adjacent agricultural fields increase interspersion and can provide essential resources, such as food and cover, needed for the occurrence of turkeys in forested locations (Porter, 2005; Rioux et al., 2009). Our findings demonstrated that the availability of open cover in an otherwise highly forested landscape was influential at both scales of inference we examined—local availability at survey stations (~814 ha) and occupancy of survey routes (~5300 ha).

Conversely, our findings in southeastern Wisconsin suggested that increased aggregation of hardwood forest cover may positively influence the occurrence of turkeys in an otherwise highly agricultural landscape. Contiguous clusters of hardwood forest patches that neighbor open‐herbaceous or agricultural fields provide a land cover mosaic for various life history needs, as forest cover provides roosting areas and male turkeys often use fields adjacent to forest edges for displaying (Wunz & Pack, 1992). A seemingly insufficient amount of forest cover likely does not directly impede occurrence of turkeys; instead, our findings further demonstrated that interspersion and configuration of forest cover confers increased probability of occupancy for turkeys within agricultural landscapes (Porter, 2005).

Effective management and conservation also require consideration of how land cover and land use changes potentially influence species distribution dynamics. In northern Wisconsin, establishment of unoccupied survey routes was negatively impacted by periods of deep snow cover (>30 cm) but positively influenced by the presence of row‐crop agriculture planted in corn within a given year. Previous studies have demonstrated that prolonged periods with deep snow restrict turkey movements (Kane et al., 2007; Porter, 1977; Roberts et al., 1995) and may lead to significant overwinter losses (Roberts et al., 1995), but fields of standing corn or residual waste corn can mitigate impacts of deep snow and influence distribution of turkeys in northern latitudes (Haroldson, 1996; Porter et al., 1980). Conversely, annual snowfall across southeast Wisconsin is typically <100 cm and persistent periods of deep snow are infrequent (Notaro et al., 2011; Wisconsin Department of Natural Resources, 2015); thus, we suggest it was unlikely that snow cover had any impact on the occurrence of turkeys in this region during our study.

In both study areas, abandonment of previously occupied routes was treated as a constant in our best‐supported models as we found no evidence that abandonment of turkeys from survey routes was explained by amount of snow cover or availability of agricultural cover. Turkey populations may yet be expanding in portions of the state; spring turkey harvest increased 17% from 2009 to 2019 in northern Wisconsin (Dhuey & Witecha, 2020), and hunter observations of turkeys while afield have also increased in the most recent decade (Rees Lohr, 2021). Additional factors may confound turkey distribution and site abandonment, such as disturbance from managed logging (Fredericksen et al., 2000) or hunting pressure. Recent studies have demonstrated that hunting activities and hunter behavior may influence male turkey movements (Gerrits et al., 2020), roosting behaviors (Wakefield et al., 2020a), and daily gobbling activity (Chamberlain et al., 2018; Wightman et al., 2019), but these conclusions have not been universal (Collier et al., 2017; Gross et al., 2015). Without a thorough understanding of the influence of hunting activities on individual turkey behaviors in our study system, we surmise that detection of a different individual during a subsequent survey would mask unavailability, whether via harvest or abandonment, of individuals from previous surveys. This unmodeled detection heterogeneity could confound our estimates (MacKenzie et al., 2006), but we note that the majority (81%) of survey routes where we encountered turkeys had detections over multiple sampling periods.

Examination of wildlife–habitat relationships across multiple spatial scales is necessary for a thorough understanding of limiting factors that influence species distributions. Recent studies have demonstrated differences in habitat associations at multiple scales of use for turkeys (Davis et al., 2017; Little et al., 2016; Pollentier et al., 2017). Our use of correlated‐replicate occupancy models to assess gobbling call‐count survey data allowed us to not only account for imperfect detection and underlying spatial autocorrelation among adjacent survey stations, but also evaluate occupancy–habitat associations at multiple scales of inference. In both of our study areas, results indicated differences in land cover characteristics that influenced probability of local availability at survey stations (~814 ha) from those that influenced probability of route occupancy (~5300 ha). Specifically, in northern Wisconsin, proximity of oak cover was a factor determining local availability, but proportion of oak cover was influential for route occupancy. In southeast Wisconsin, local availability was influenced by large patches of row‐crop agriculture and interspersion of upland hardwoods, whereas route occupancy appeared to be predominately affected by aggregation of available upland hardwood forest cover.

We note, however, that the proportion of open cover was highly influential at both the survey station and route scales for turkeys in heavily forested northern Wisconsin. Additionally, even though specific land cover characteristics differed between scales of inference in southeast Wisconsin, we suggest that perhaps these metrics were ecologically similar for a habitat generalist like the turkey and inferred that interspersion and aggregation of forest cover in an agricultural landscape was important at both spatial scales. Extent and grain contribute to our understanding of wildlife–habitat associations across different spatial scales (Hobbs, 2003; Wiens, 1989); perhaps differences in grain between our sampling units (survey stations [~814 ha] and survey routes [~5300 ha]) were not great enough to discern different land cover attributes for turkeys at those scales we considered. Variances in habitat associations among scales can be difficult to determine in homogeneous landscapes (Schaefer & Messier, 1995) like those we studied. Conversely, our findings demonstrated that interspersion and aggregations of contrasting cover types in otherwise predominately forested or open‐agricultural landscapes may influence distribution and likelihood of occurrence for turkeys at multiple scales of inference. We suggest that consistent habitat association patterns across spatial scales represent those attributes that are of fundamental importance to the distribution and occurrence of turkeys in northern and midwestern landscapes. The advantage of examining multiple scales of inference, whether different attributes occur across scales or not, is that it enables managers to identify, focus, and monitor ecological costs and benefits of management and conservation decisions for wildlife (Ciarniello et al., 2007; Levin, 1992). Decisions based on only one scale of inference are likely limited in their scope and could result in poor or unintended management outcomes (Guisan & Thuiller, 2005; Jackson & Fahrig, 2015; Kotliar & Wiens, 1990).

## CONFLICT OF INTEREST

The authors have no conflicts of interest to declare.

## AUTHOR CONTRIBUTIONS

**Christopher D. Pollentier:** Conceptualization (equal); Data curation (lead); Formal analysis (lead); Funding acquisition (lead); Investigation (equal); Methodology (equal); Project administration (lead); Resources (lead); Supervision (lead); Visualization (lead); Writing – original draft (lead); Writing – review & editing (equal). **Michael A. Hardy:** Conceptualization (equal); Data curation (supporting); Formal analysis (supporting); Investigation (equal); Methodology (equal); Writing – original draft (supporting); Writing – review & editing (equal). **R. Scott Lutz:** Conceptualization (equal); Formal analysis (equal); Funding acquisition (supporting); Investigation (equal); Methodology (equal); Writing – review & editing (equal). **Scott D. Hull:** Conceptualization (equal); Funding acquisition (supporting); Investigation (equal); Methodology (equal); Project administration (supporting); Resources (supporting); Supervision (supporting); Writing – review & editing (equal). **Benjamin Zuckerberg:** Conceptualization (equal); Methodology (equal); Writing – review & editing (equal).

## Data Availability

PRESENCE input files, including detection–nondetection histories, survey‐specific environmental data, and station‐ and route‐specific land cover metrics from FRAGSTATS, have been deposited in the Dryad digital repository (https://doi.org/10.5061/dryad.3bk3j9km2).

## References

[ece38419-bib-0001] Arnold, T. W. (2010). Uninformative parameters and model selection using Akaike’s information criterion. Journal of Wildlife Management, 74, 1175–1178. 10.1111/j.1937-2817.2010.tb01236.x

[ece38419-bib-0002] Backs, S. E. , & Eisfelder, C. H. (1990). Criteria and guidelines for wild turkey release priorities in Indiana. Proceedings of the National Wild Turkey Symposium, 6, 134–143.

[ece38419-bib-0003] Bailey, L. L. , Hines, J. E. , Nichols, J. D. , & MacKenzie, D. I. (2007). Sampling design trade‐offs in occupancy studies with imperfect detection: Examples and software. Ecological Applications, 17, 281–290. 17479851 10.1890/1051-0761(2007)017[0281:sdtios]2.0.co;2

[ece38419-bib-0004] Bailey, L. L. , MacKenzie, D. I. , & Nichols, J. D. (2014). Advances and applications of occupancy models. Methods in Ecology and Evolution, 5, 1269–1279. 10.1111/2041-210X.12100

[ece38419-bib-0005] Bevill, W. V. (1973). Some factors influencing gobbling activity among turkeys. Proceedings of the Southeastern Association of Game and Fish Commissioners, 27, 62–73.

[ece38419-bib-0006] Bevill, W. V. (1975). Setting spring gobbler hunting seasons by timing peak gobbling. Proceedings of the National Wild Turkey Symposium, 3, 198–204.

[ece38419-bib-0007] Boncoraglio, G. , & Saino, N. (2007). Habitat structure and the evolution of bird song: A meta‐analysis of the evidence for the acoustic adaptation hypothesis. Functional Ecology, 21, 134–142. 10.1111/j.1365-2435.2006.01207.x

[ece38419-bib-0008] Burnham, K. P. , & Anderson, D. R. (2002). Model selection and multimodel inference: A practical information‐theoretic approach, 2nd edn. Springer.

[ece38419-bib-0009] Chamberlain, M. J. , Wightman, P. H. , Cohen, B. S. , & Collier, B. A. (2018). Gobbling activity of eastern wild turkeys relative to male movements and female nesting phenology in South Carolina. Wildlife Society Bulletin, 42, 632–642. 10.1002/wsb.932

[ece38419-bib-0010] Chapagain, B. P. , Poudyal, N. C. , Joshi, O. , Watkins, C. , & Applegate, R. D. (2020). Seasonal and regional differences in economics benefits of turkey hunting. Wildlife Society Bulletin, 44, 271–280. 10.1002/wsb.1093

[ece38419-bib-0011] Chavez, C. (2014). Occupancy and habitat association for eastern wild turkey (*Meleagris gallopavo silvestris*) in east Texas (144 pp). M.Sc. thesis. Stephen F. Austin State University.

[ece38419-bib-0012] Ciarniello, L. M. , Boyce, M. S. , Seip, D. R. , & Heard, D. C. (2007). Grizzly bear habitat selection is scale dependent. Ecological Applications, 17, 1424–1440. 10.1890/06-1100.1 17708219

[ece38419-bib-0013] Collier, B. A. , Wightman, P. , Chamberlain, M. J. , Cantrell, J. , & Ruth, C. (2017). Hunting activity and male wild turkey movements in South Carolina. Journal of the Southeastern Association of Fish and Wildlife Agencies, 4, 85–93.

[ece38419-bib-0014] Courlas, J. C. (2014). Investigating the influence of landscape composition and pattern on spring habitat use in an introduced wild turkey population in the northern Great Plains (80 pp). M.sc. thesis. University of Wisconsin‐Madison.

[ece38419-bib-0015] Davis, A. , Wang, G. , Martin, J. , Belant, J. , Butler, A. , Rush, S. , & Godwin, D. (2017). Landscape‐abundance relationships of male eastern wild turkeys *Meleagris gallopavo silvestris* in Mississippi, USA. Acta Ornithologica, 52, 127–139. 10.3161/00016454AO2017.52.2.001

[ece38419-bib-0016] DeYoung, C. A. , & Priebe, J. C. (1987). Comparison of inventory methods for wild turkeys in south Texas. Proceedings of the Southeastern Association of Fish and Wildlife Agencies, 41, 294–298.

[ece38419-bib-0017] Dhuey, B. , & Witecha, M. (2020). Spring turkey harvest report, 2019. In J. Kitchell , & B. Dhuey (Eds.), Wisconsin Wildlife Surveys. Wisconsin Department of Natural Resources. https://dnr.wisconsin.gov/topic/WildlifeHabitat/reports.html

[ece38419-bib-0018] Dormann, C. F. , Elith, J. , Bacher, S. , Buchmann, C. , Carl, G. , Carré, G. , García Marquéz, J. R. , Gruber, B. , Lafourcade, B. , Leitāo, P. J. , Münkemuller, T. , McClean, C. , Osborne, P. E. , Reineking, B. , Schröder, B. , Skidmore, A. K. , Zurell, D. , & Lautenbach, S. (2013). Collinearity: A review of methods to deal with it and a simulation study evaluating their performance. Ecography, 36, 27–46. 10.1111/j.1600-0587.2012.07348.x

[ece38419-bib-0019] Ewers, R. M. , & Didham, R. K. (2006). Confounding factors in the detection of species responses to habitat fragmentation. Biological Reviews, 81, 117–142. 10.1017/S1464793105006949 16318651

[ece38419-bib-0020] Ey, E. , & Fischer, J. (2009). The “acoustic adaptation hypothesis”–a review of the evidence from birds, anurans, and mammals. Bioacoustics, 19, 21–48. 10.1080/09524622.2009.9753613

[ece38419-bib-0021] Fahrig, L. (2003). Effects of habitat fragmentation on biodiversity. Annual Review of Ecology, Evolution, and Systematics, 34, 487–515. 10.1146/annurev.ecolsys.34.011802.132419

[ece38419-bib-0022] Field, S. A. , Tyre, A. J. , & Possingham, H. P. (2005). Optimizing allocation of monitoring effort under economic and observational constraints. Journal of Wildlife Management, 69, 473–482.

[ece38419-bib-0023] Fredericksen, T. S. , Ross, B. D. , Hoffman, W. , Ross, E. , Morrison, M. L. , Beyea, J. , Lester, M. B. , & Johnson, B. N. (2000). The impact of logging on wildlife: A study in northeastern Pennsylvania. Journal of Forestry, 98(4), 4–10.

[ece38419-bib-0024] Gerrits, A. P. , Wightman, P. H. , Cantrell, J. R. , Ruth, C. , Chamberlain, M. J. , & Collier, B. A. (2020). Movement ecology of spring wild turkey hunters on public lands in South Carolina, USA. Wildlife Society Bulletin, 44, 260–270. 10.1002/wsb.1094

[ece38419-bib-0025] Glennon, M. J. , & Porter, W. F. (1999). Using satellite imagery to assess landscape‐scale habitat for wild turkeys. Wildlife Society Bulletin, 27, 646–653.

[ece38419-bib-0026] Gross, J. T. , Cohen, B. S. , Collier, B. A. , & Chamberlain, M. J. (2015). Influences of hunting on movements of male wild turkeys during spring. National Wild Turkey Symposium, 11, 259–268.

[ece38419-bib-0027] Guisan, A. , & Thuiller, W. (2005). Predicting species distribution: offering more than simple habitat models. Ecology Letters, 8, 993–1009. 10.1111/j.1461-0248.2005.00792.x 34517687

[ece38419-bib-0028] Gustafson, E. J. , Parker, G. R. , & Backs, S. E. (1994). Evaluating spatial pattern of wildlife habitat: A case study of the wild turkey (*Meleagris gallopavo*). American Midland Naturalist, 131, 24–33. 10.2307/2426605

[ece38419-bib-0029] Haroldson, K. J. (1996). Energy requirements for winter survival of wild turkeys. Proceedings of the National Wild Turkey Symposium, 7, 9–14.

[ece38419-bib-0030] Hayes, D. B. , & Monfils, M. J. (2015). Occupancy modeling of bird point counts: Implications of mobile animals. Journal of Wildlife Management, 79, 609–628. 10.1002/jwmg.943

[ece38419-bib-0031] Healy, W. M. (1992). Population influences: Environment. In J. G. Dickson (Ed.), The wild turkey: Biology and management (pp. 129–143). Stackpole Books.

[ece38419-bib-0032] Healy, W. M. , & Powell, S. M. (1999). Wild turkey harvest management: Biology, strategies, and techniques. U.S. Fish and Wildlife Service, Biological Technical Publication.

[ece38419-bib-0033] Hines, J. E. (2006). PRESENCE v12.23: Software to estimate patch occupancy and related parameters. U.S. Geological Survey Patuxent Wildlife Research Center. http://mbr‐pwrc.usgs.gov/software/presence.html

[ece38419-bib-0034] Hines, J. E. , Nichols, J. D. , & Collazo, J. A. (2014). Multiseason occupancy models for correlated replicate surveys. Methods in Ecology and Evolution, 5, 583–591. 10.1111/2041-210X.12186

[ece38419-bib-0035] Hines, J. E. , Nichols, J. D. , Royle, J. A. , MacKenzie, D. I. , Gopalaswamy, A. M. , Kumar, N. S. , & Karanth, K. U. (2010). Tigers on trails: occupancy modeling for cluster sampling. Ecological Applications, 20, 1456–1466. 10.1890/09-0321.1 20666261

[ece38419-bib-0036] Hobbs, N. T. (2003). Challenges and opportunities in integrating ecological knowledge across scales. Forest Ecology and Management, 181, 223–238. 10.1016/S0378-1127(03)00135-X

[ece38419-bib-0037] Hoeting, J. A. (2009). The importance of accounting for spatial and temporal correlation in analyses of ecological data. Ecological Applications, 19, 574–577. 10.1890/08-0836.1 19425418

[ece38419-bib-0038] Hoffman, R. W. (1990). Chronology of gobbling and nesting activities of Merriam’s wild turkeys. Proceedings of the National Wild Turkey Symposium, 6, 25–31.

[ece38419-bib-0039] Isabelle, J. L. , Butler, A. B. , Ruth, C. , & Lowrey, D. K. (2018). Considerations for timing of spring wild turkey hunting seasons in the southeastern United States. Journal of the Southeastern Association of Fish and Wildlife Agencies, 5, 106–113.

[ece38419-bib-0040] Jackson, H. B. , & Fahrig, L. (2015). Are ecologists conducting research at the optimal scale? Global Ecology and Biogeography, 24, 52–63. 10.1111/geb.12233

[ece38419-bib-0041] Kane, D. F. , Kimmel, R. O. , & Faber, W. E. (2007). Winter survival of wild turkey females in central Minnesota. Journal of Wildlife Management, 71, 1800–1807. 10.2193/2006-008

[ece38419-bib-0042] Kéry, M. , Gardner, B. , & Monnerat, C. (2010). Predicting species distributions from checklist data using site‐occupancy models. Journal of Biogeography, 37, 1851–1862. 10.1111/j.1365-2699.2010.02345.x

[ece38419-bib-0043] Kienzler, J. M. , Little, T. W. , & Fuller, W. A. (1996). Effects of weather, incubation, and hunting on gobbling activity in wild turkeys. Proceedings of the National Wild Turkey Symposium, 7, 61–67.

[ece38419-bib-0044] Kotliar, N. B. , & Wiens, J. A. (1990). Multiple scales of patchiness and patch structure: A hierarchical framework for the study of heterogeneity. Oikos, 59, 253–260. 10.2307/3545542

[ece38419-bib-0045] Kubisiak, J. F. , Rolley, R. E. , Paisley, R. N. , & Wright, R. G. (2001). Wild turkey: Ecology and management in Wisconsin (44 pp.). Wisconsin Department of Natural Resources.

[ece38419-bib-0046] Kurzejeski, E. W. , & Lewis, J. B. (1985). Application of PATREC modeling to wild turkey management in Missouri. Proceedings of the National Wild Turkey Symposium, 5, 269–284.

[ece38419-bib-0047] Lavoie, M. , Blanchette, P. , Larivière, S. , & Tremblay, J.‐P. (2017). Winter and summer weather modulate the demography of wild turkeys at the northern edge of the species distribution. Population Ecology, 59, 239–249. 10.1007/s10144-017-0585-2

[ece38419-bib-0048] Legendre, P. (1993). Spatial autocorrelation: trouble or new paradigm? Ecology, 74, 1659–1673. 10.2307/1939924

[ece38419-bib-0049] Levin, S. A. (1992). The problem of pattern and scale in ecology. Ecology, 73, 1943–1967. 10.2307/1941447

[ece38419-bib-0050] Lint, J. R. , Leopold, B. D. , & Hurst, G. A. (1995). Comparison of abundance indexes and population estimates for wild turkey gobblers. Wildlife Society Bulletin, 23, 164–168.

[ece38419-bib-0051] Little, A. R. , Chamberlain, M. J. , Conner, L. M. , & Warren, R. J. (2016). Habitat selection of wild turkeys in burned longleaf pine savannas. Journal of Wildlife Management, 80, 1280–1289. 10.1002/jwmg.21114

[ece38419-bib-0052] Little, T. W. (1980). Wild turkey restoration in “marginal” Iowa habitats. Proceedings of the National Wild Turkey Symposium, 4, 45–60.

[ece38419-bib-0053] MacKenzie, D. I. , Nichols, J. D. , Hines, J. E. , Knutson, M. G. , & Franklin, A. B. (2003). Estimating site occupancy, colonization, and local extinction when a species is detected imperfectly. Ecology, 84, 2200–2207. 10.1890/02-3090

[ece38419-bib-0054] MacKenzie, D. I. , Nichols, J. D. , Lachman, G. B. , Droege, S. , Royle, J. A. , & Langtimm, C. A. (2002). Estimating site occupancy rates when detection probabilities are less than one. Ecology, 83, 2248–2255.

[ece38419-bib-0055] MacKenzie, D. I. , Nichols, J. D. , Royle, J. A. , Pollock, K. H. , Bailey, L. L. , & Hines, J. E. (2006). Occupancy estimation and modeling: Inferring patterns and dynamics of species occurrence. Elsevier.

[ece38419-bib-0056] MacKenzie, D. I. , & Royle, J. A. (2005). Designing occupancy studies: General advice and allocating survey effort. Journal of Applied Ecology, 42, 1105–1114. 10.1111/j.1365-2664.2005.01098.x

[ece38419-bib-0057] Marable, M. K. , Belant, J. L. , Godwin, D. , & Wang, G. (2012). Effects of resource dispersion and site familiarity on movements of translocated wild turkeys on fragmented landscapes. Behavioural Processes, 91, 119–124. 10.1016/j.beproc.2012.06.006 22750280

[ece38419-bib-0058] McGarigal, K. , Cushman, S. A. , & Ene, E. (2012). FRAGSTATS v4: Spatial pattern analysis program for categorical and continuous maps. https://www.umass.edu/landeco/research/fragstats/fragstats.html

[ece38419-bib-0059] Morin, D. J. , Yackulic, C. B. , Diffendorfer, J. E. , Lesmeister, D. B. , Nielsen, C. K. , Reid, J. , & Schauber, E. M. (2020). Is your ad hoc model selection strategy affecting your multimodel inference? Ecosphere, 11, 1–16. 10.1002/ecs2.2997

[ece38419-bib-0060] National Operational Hydrologic Remote Sensing Center (NOHRSC) (2004). Snow Data Assimilation System (SNODAS) data products at National Snow and Ice Data Center (NSIDC), version 1. National Snow and Ice Data Center.

[ece38419-bib-0061] Nichols, J. D. , Thomas, L. , & Conn, P. B. (2009). Inferences about landbird abundance from count data: Recent advances and future directions. In D. L. Thomson , E. G. Cooch , & M. J. Conroy (Eds.), Modeling demographic processes in marked populations (pp. 201–235). Springer.

[ece38419-bib-0062] Niedzielski, B. , & Bowman, J. (2015). Survival and cause‐specific mortality of the female eastern wild turkey at its northern range edge. Wildlife Research, 41, 545–551. 10.1071/WR14061

[ece38419-bib-0063] Notaro, M. , Lorenz, D. J. , Vimont, D. , Vavrus, S. , Kucharik, C. , & Franz, K. (2011). 21st century Wisconsin snow projections based on an operational snow model driven by statistically downscaled climate data. International Journal of Climatology, 31, 1615–1633. 10.1002/joc.2179

[ece38419-bib-0064] Ogden, L. E. (2015). Return of the native. New Scientist, 228, 44–45. 10.1016/S0262-4079(15)31714-0

[ece38419-bib-0065] Pagano, A. M. , & Arnold, T. W. (2009). Detection probabilities for ground‐based breeding waterfowl surveys. Journal of Wildlife Management, 73, 392–398. 10.2193/2007-411

[ece38419-bib-0066] Paisley, R. N. , Conrad, P. J. , Denk, D. D. , & Kubisiak, J. F. (2000). Home range characteristics of eastern wild turkey gobblers in Wisconsin’s driftless region. Wisconsin Department of Natural Resources. https://wi‐dnr.widencollective.com/c/yxkyrwsu

[ece38419-bib-0067] Paisley, R. N. , Wright, R. G. , & Kubisiak, J. F. (1996). Use of agricultural habitats and foods by wild turkeys in southwestern Wisconsin. Proceedings of the National Wild Turkey Symposium, 7, 69–73.

[ece38419-bib-0068] Palmer, W. E. , Hurst, G. A. , & Lint, J. R. (1990). Efforts, success, and characteristics of spring turkey hunters on Tallahala Wildlife Management Area, Mississippi. Proceedings of the National Wild Turkey Symposium, 6, 208–213.

[ece38419-bib-0069] Pollentier, C. D. , Hardy, M. A. , Lutz, R. S. , & Hull, S. D. (2019). Correlated‐replicate occupancy models for wild turkey gobbling call‐count surveys. Wildlife Society Bulletin, 43, 515–526. 10.1002/wsb.987

[ece38419-bib-0070] Pollentier, C. D. , Hardy, M. A. , Lutz, R. S. , Hull, S. D. , & Zuckerberg, B. (2021). Data from: Gobbling across landscapes: Eastern wild turkey distribution and occupancy‐habitat associations. *Dryad Digital Repository*, Dataset, 10.5061/dryad.3bk3j9km2 PMC8717345 35003671

[ece38419-bib-0071] Pollentier, C. D. , Lutz, R. S. , & Drake, D. (2017). Female wild turkey habitat selection in mixed forest‐agricultural landscapes. Journal of Wildlife Management, 81, 487–497. 10.1002/jwmg.21214

[ece38419-bib-0072] Porter, W. F. (1977). Home range dynamics of wild turkeys in southeastern Minnesota. Journal of Wildlife Management, 41, 434–437. 10.2307/3800512

[ece38419-bib-0073] Porter, W. F. (1992). Habitat requirements. In J. G. Dickson (Ed.), The wild turkey: Biology and management (pp. 202–213). Stackpole Books.

[ece38419-bib-0074] Porter, W. F. (2005). Understanding the ecology of wild turkeys in northern latitudes. Proceedings of the National Wild Turkey Symposium, 9, 307–313.

[ece38419-bib-0075] Porter, W. F. , & Ludwig, J. R. (1980). Use of gobbling counts to monitor the distribution and abundance of wild turkeys. Proceedings of the National Wild Turkey Symposium, 4, 61–68.

[ece38419-bib-0076] Porter, W. F. , Tangen, R. D. , Nelson, G. C. , & Hamilton, D. A. (1980). Effects of corn food plots on wild turkeys in the upper Mississippi Valley. Journal of Wildlife Management, 44, 456–462. 10.2307/3807977

[ece38419-bib-0077] Rees Lohr, J. (2021). Deer hunter wildlife survey, 2020. In J. Kitchell , & B. Dhuey (Eds.), Wisconsin Wildlife Surveys. Wisconsin Department of Natural Resources. https://dnr.wisconsin.gov/topic/WildlifeHabitat/reports.html

[ece38419-bib-0078] Rioux, S. , Bélisle, M. , & Giroux, J.‐F. (2009). Effects of landscape structure on male density and spacing patterns in wild turkeys (*Meleagris gallopavo*) depend on winter severity. The Auk, 126, 673–683. 10.1525/auk.2009.08127

[ece38419-bib-0079] Roberts, S. D. , Coffey, J. M. , & Porter, W. F. (1995). Survival and reproduction of female turkeys in New York. Journal of Wildlife Management, 59, 437–447. 10.2307/3802449

[ece38419-bib-0080] Rota, C. T. , Fletcher, R. J. Jr , Dorazio, R. M. , & Betts, M. G. (2009). Occupancy estimation and the closure assumption. Journal of Applied Ecology, 46, 1173–1181. 10.1111/j.1365-2664.2009.01734.x

[ece38419-bib-0081] Schaefer, J. A. , & Messier, F. (1995). Habitat selection as a hierarchy: the spatial scales of winter foraging by muskoxen. Ecography, 18, 333–344. 10.1111/j.1600-0587.1995.tb00136.x

[ece38419-bib-0082] Scott, V. E. , & Boeker, E. L. (1972). An evaluation of wild turkey call counts in Arizona. Journal of Wildlife Management, 36, 628–630. 10.2307/3799097

[ece38419-bib-0083] United States Fish and Wildlife Service (2016). 2016 National survey of fishing, hunting, and wildlife‐associated recreation. https://www.census.gov/content/dam/Census/library/publications/2018/demo/fhw16‐nat.pdf

[ece38419-bib-0084] USDA National Agricultural Statistics Service (2017). Wisconsin Cropland Data Layer, 2013–2017. United States Department of Agriculture. https://nassgeodata.gmu.edu/CropScape

[ece38419-bib-0085] Wakefield, C. T. , Martin, J. A. , Wightman, P. H. , Bond, B. T. , Lowery, D. K. , Cohen, B. S. , Collier, B. A. , & Chamberlain, M. J. (2020a). Hunting activity effects on roost selection by male wild turkeys. Journal of Wildlife Management, 84, 458–467. 10.1002/jwmg.21812

[ece38419-bib-0086] Wakefield, C. T. , Martin, J. A. , Wightman, P. H. , Bond, B. T. , Lowery, D. K. , Cohen, B. S. , Collier, B. A. , & Chamberlain, M. J. (2020b). Hunting and nesting phenology influence gobbling of wild turkeys. Journal of Wildlife Management, 84, 448–457. 10.1002/jwmg.21804

[ece38419-bib-0087] Whittington, J. , Heuer, K. , Hunt, B. , Hebblewhite, M. , & Lukacs, P. M. (2015). Estimating occupancy using spatially and temporally replicated snow surveys. Animal Conservation, 18, 92–101. 10.1111/acv.12140

[ece38419-bib-0088] Wiens, J. A. (1989). Spatial scaling in ecology. Functional Ecology, 3, 385–397. 10.2307/2389612

[ece38419-bib-0089] Wightman, P. H. , Kilgo, J. C. , Vukovich, M. , Cantrell, J. R. , Ruth, C. R. , Cohen, B. S. , Chamberlain, M. J. , & Collier, B. A. (2019). Gobbling chronology of eastern wild turkeys in South Carolina. Journal of Wildlife Management, 83, 325–333. 10.1002/jwmg.21600

[ece38419-bib-0090] Wisconsin Department of Natural Resources (2015). Ecological landscapes of Wisconsin: An assessment of ecological resources and a guide to planning sustainable management. Wisconsin Department of Natural Resources. https://dnr.wisconsin.gov/topic/Lands/Book.html

[ece38419-bib-0091] Wisconsin Department of Natural Resources (2016). Wiscland 2 land cover user guide. Wisconsin Department of Natural Resources. https://dnr.wisconsin.gov/maps/WISCLAND

[ece38419-bib-0092] Wunz, G. A. , & Hayden, A. H. (1975). Winter mortality and supplemental feeding of turkeys in Pennsylvania. Proceedings of the National Wild Turkey Symposium, 3, 61–69.

[ece38419-bib-0093] Wunz, G. A. , & Pack, J. C. (1992). Eastern turkey in eastern oak‐hickory and northern hardwood forests. In J. G. Dickson (Ed.), The wild turkey: Biology and management (pp. 232–264). Stackpole Books.

